# Bias in adjudication: Investigating the impact of artificial intelligence, media, financial and legal institutions in pursuit of social justice

**DOI:** 10.1371/journal.pone.0315270

**Published:** 2025-01-03

**Authors:** Kashif Javed, Jianxin Li

**Affiliations:** School of Law, Zhengzhou University, Zhengzhou, Henan, China; Hainan University, CHINA

## Abstract

The latest global progress report highlights numerous challenges in achieving justice goals, with bias in artificial intelligence (AI) emerging as a significant yet underexplored issue. This paper investigates the role of AI in addressing bias within the judicial system to promote equitable social justice. Analyzing weekly data from January 1, 2019, to December 31, 2023, through wavelet quantile correlation, this study examines the short, medium, and long-term impacts of integrating AI, media, international legal influence (ILI), and international financial institutions (IFI) as crucial factors in achieving Sustainable Development Goal 16 (SDG-16), which focuses on justice. The findings indicate that AI, media, ILI, and IFI can help reduce bias in the medium and long term, although their effects appear mixed and less significant in the short term. Our research proposes a comprehensive policy framework that addresses the complexities of implementing these technologies in the judicial system. We conclude that successfully integrating AI requires a supportive global policy environment that embraces technological innovation, financial backing, and robust regulation to prevent potential disruptions that could reinforce inequalities, perpetuate structural injustices, and exacerbate human rights issues, ultimately leading to more biased outcomes in social justice.

## 1. Introduction

Justice plays a pivotal role in the smooth functioning of society by providing a reasonable framework that fosters fairness, guards fundamental rights, and preserves social order. Judicial adjudication is the process that is adopted by courts to settle disputes in a society. It ultimately serves the goal of justice by providing a peaceful resolution of societal disputes. Therefore, legal adjudication is crucial in maintaining peace and tranquility, upholding norms, and fostering societal development. As the world population surpasses 8 billion, judicial adjudication faces challenges such as a shortage of judges and delayed justice, reflecting a compromised delivery of justice in today’s global sustainability agenda [1]. The interconnection between justice and sustainability is pivotal; sustainable development goals (SDGs), particularly SDG 16, emphasize promoting peaceful and inclusive societies, providing access to justice for all, and building effective institutions. This goal highlights the necessity of integrating justice into sustainability efforts globally [2]. The sustainable development report illustrates the challenges in achieving SDG 16, underscoring the varying levels of progress and substantial obstacles many countries face in establishing peace, justice, and strong institutions [3]. It also denotes the declining confidence of the general populace in the judicial system, thereby raising concerns about the institution’s legitimacy. In this scenario, artificial intelligence (AI) is promising to solve the problems of judiciaries by making processes smoother and quicker and helping to eliminate backlogs, hence enhancing the accessibility of justice to more people. As Artificial intelligence makes its way into the legal and judicial fields, it is changing the mechanisms of how justice is exercised and delivered. Various AI tools are now employed in several areas of law practice, including legal research, predicting the probabilities of success of a legal action, and case management, which determines the suitability of a sentence or parole [4].

These technologies can accelerate processes and make the judicial system more accessible and efficient [5]. But, as AI systems like ROSS Intelligence and IBM’s Watson begin to play a role in legal adjudication, it is essential to consider their impact on the fairness and transparency of the judicial process along with the potential for bias in such tools becomes an important consideration [6]. AI bias can originate from several causes, including flaws in data introduced during training or biases in the manner in which AI algorithms analyze and score information. If mismanaged, biased AI may result in biasing outcomes that are primarily negative to the already marginalized communities and only serve to deepen these inequities rather than improve them. This risk highlights the necessity of upholding AI systems to higher standards of fairness and accountability, especially in legal adjudication areas [7].

The possibility of bias within AI systems represents a direct threat to SDG 16 as it encompasses issues of justice, fairness, and inclusion. When AI algorithms propagate or introduce bias, such efforts are nullified as these constrain the availability of justice to the masses and can lead to inappropriately determined sentences, biased law enforcement, and discrimination in legal adjudication. This is just one of the many ramifications that society can endure since bias in AI technology can lead to institutionalized forms of discrimination against certain ethnicities, classes, reproduction, or other minorities, as was evident in the case of using COMPASS [8]. For example, suppose an AI-based risk prediction model used in the criminal justice system prejudices certain demographics because the training data in history is biased. In that case, it will lead to excessive sentences, additional sentencing requirements, or requirements for conditions on parole. Because of these biases, there is a loss of confidence in the citizens in legal institutions [9].

AI’s potential to improve judicial efficiency must be balanced with the risks it poses regarding bias and discrimination. In our econometric model, AI is indeed the core variable of interest, as it represents the transformative potential of technology within the justice system. Media, ILI (International Legal Institution), and IFI (International Financial Institutions) serve as supportive variables that help contextualize the impact of AI. Their inclusion allows for a more comprehensive understanding of the factors that can enhance or inhibit the effective implementation of AI technologies in justice processes. The interconnectedness between justice systems and variables such as media, Artificial Intelligence (AI), international legal institutions, and international financial institutions reveals a complex interplay that shapes legal outcomes and public perceptions. Media influences public trust by highlighting legal processes and systemic issues, while AI enhances efficiency and accessibility within justice systems, albeit raising concerns about bias and fairness. International legal institutions set global standards that national systems may adopt, promoting consistency and accountability. In contrast, international financial institutions often provide funding for judicial reforms, impacting the effectiveness and integrity of these systems.

AI could also introduce bias and discrimination into social justice and adjudication. The lack of transparency in AI systems could affect the due process of law. Furthermore, the media may also influence judicial systems by shaping public perceptions and monitoring judicial decisions. Comprehensive interviews with the Czech Constitutional Court have confirmed that the media can affect judges’ decision-making processes [10]. Additionally, international financial institutions like the World Bank, International Monetary Fund (IMF), and the Asian Development Bank, as well as international legal institutions such as the International Court of Justice (ICJ) and UN conventions, could also impact judicial systems [11]. Therefore, examining the influence of AI alongside media, international financial institutions (IFI), and international legal influence (ILI) is rational. Together, these elements create a dynamic ecosystem where improvements in one area can significantly influence the others, illustrating the multifaceted nature of justice in a globalized context.

The above discourse induces the hypothesis that AI might play a critical role in addressing the recent challenges faced by judicial adjudication. We can also hypothesize that media, ILI, and IFI are also helpful to AI in bridging the gaps in promoting justice in society to lead to sustainable development. Therefore, it is rational to assume that the disruption exerted by AI bias might be dynamic, and thus, it can affect the global judicial system. The recent study’s central point is exploring these issues, and therefore, we postulate the following query:

**Research question**: How does artificial intelligence affect bias in the judicial system alongside the influence of media, international legal institutions, and international financial institutions?

In the context of the above query, our study extends the previous literature in the following ways. First, to the best of our modest understanding, this study introduces AI as a novel driver to measure how it can assist in handling bias in judicial adjudication for justice and sustainability. Second, the study extends the pertinent literature by empirically testifying to the legitimacy theory and bias. This study, therefore, aims to rigorously assess the impacts of AI integration within judicial systems at a global level, employing advanced methodologies such as the wavelet-quantile correlation approach to ensure the validity and reliability of the findings [12]. By doing so, the research contributes to understanding the potential benefits and risks of AI in justice systems and aids in developing comprehensive policies to enhance justice in alignment with global sustainability goals.

Therefore, this article examines SDG 16’s sub-goal 16.3, which promotes the rule of law and equal access to justice for all. It highlights the importance of judicial adjudication in ensuring fair justice and explores the integration of artificial intelligence (AI) to improve judicial efficiency. However, it warns that AI could introduce biases that undermine justice and inclusivity, key components of SDG 16.3. Additionally, the article addresses sub-goal 16.6, which focuses on developing effective, accountable, and transparent institutions. It emphasizes the need for fairness and transparency in AI systems used in judicial processes. While AI has the potential to enhance access and institutional transparency, careful implementation is essential to prevent perpetuating existing inequalities and biases in the justice system. The remaining portions of the research are further broken down into the following sections: A review of the most current relevant literature is presented in Section 2, followed by a description of the theoretical foundation of the testable model in Section 3, an outline of the econometric methodology in Section 4, a report and discussion of the results in Section 5, and a conclusion to the study with policy implications in Section 6.

## 2. Literature review

Technology has some natural aspects, such as food and shelter, which make us stay safe. Vehicles have made us approach most corners of the world, and even now, the landing on the Moon and the planning of Mars are no longer myths. Similarly, modern computers have extended our brains, and replicas of brains have become possible, i.e., artificial intelligence. Hence, technology has become a symbol of modern humans. However, there are three types of debates about the philosophy of technology in society. One approach considers technology as an independent force that directs society [13]. Others consider it a human-dependent force, and some consider it interdependent as technology and society have evolved simultaneously. Prima facie, the last approach is the interdependency of society, technology, and co-evolution. The principle of "Salus Populi Suprema Lex Est," or "The welfare of the people shall be the supreme law," serves as a foundational tenet in legal philosophy that emphasizes the primacy of public welfare in the formulation and application of laws. This principle is particularly relevant in discussions of justice within the context of technology-driven judicial systems. Artificial intelligence is vital and valuable to meet the modern needs of an enlightened society. We may reach new horizons by using AI technology and techniques to access justice, as a just society can only prevail with harmony and peace on earth.

As AI technologies become increasingly integrated into legal processes, it is crucial to assess how they align with the fundamental goal of serving public welfare. This alignment is particularly relevant in the context of the fourth industrial revolution, often referred to as the AI revolution. The interdependency of society, technology, and co-evolution is a compelling framework for understanding these dynamics [14]. Historically, the fascination with AI emerged from its potential to perform diverse tasks, as illustrated in Lawlor’s early discussions about the capabilities of computers [15]. This initial excitement was echoed in Herbert Simon’s predictions that AI would surpass human performance in various domains. This reality materialized in 1997 when IBM’s Deep Blue famously defeated world chess champion Garry Kasparov [16].

In this landscape, the theory of distributive justice, which focuses on the equitable distribution of resources and benefits within society, becomes particularly pertinent. When implemented responsibly, AI technology can facilitate the realization of distributive justice by identifying disparities in judicial outcomes and ensuring fair treatment across diverse demographics. For example, AI-driven data analysis can uncover biases in sentencing patterns, prompting judicial reforms that align with equitable principles [17]. Furthermore, procedural justice theory underscores the importance of fair processes in legal proceedings. AI systems can enhance procedural fairness by offering transparent and consistent frameworks for decision-making. However, a significant concern arises from the opacity of algorithms, which can obscure the decision-making process of affected parties. Maintaining public trust in the legal system necessitates that individuals understand how decisions are made [18].

Additionally, restorative justice aims to repair harm and promote reconciliation between offenders and victims, a process that AI technologies can support through improved communication and mediation. Nonetheless, ethical dilemmas surface when AI systems prioritize efficiency over empathy, potentially undermining the relational aspects that are central to restorative practices [19]. Thus, while AI holds promise for advancing various theories of justice within legal contexts, it also raises critical questions about its ethical application and the preservation of human values in judicial processes. Integrating distributive and procedural justice theories can shed light on the impact of artificial intelligence (AI) on judicial processes. Distributive justice involves fair allocation of resources and outcomes, in contrast, procedural justice focuses on the fairness. AI can improve efficiency and reduce case backlogs, resulting in faster access to justice for a larger population. AI has also the potential to support procedural justice by speeding up legal procedures and making them more accessible. However, if AI systems are biased or lack proper oversight, the implications can be significant.

### 2.1 Judicial system and sustainable development goal-16

A recent study highlights the critical link between justice and Sustainable Development Goal 16, emphasizing the importance of robust legal frameworks and equitable governance in promoting sustainable development. Kopainsky [20] advocate for incorporating justice frameworks into development programs to improve fairness. Burdon & Martel [21] show how inclusive legal systems can reduce inequities and increase community participation in governance. According to Satterthwaite & Dhital [22], justice is critical for achieving all SDGs, emphasizing the importance of analytical approaches that prioritize access to justice. Bennett et al. [23] offer a comparative view, exposing how different governance models influence justice and sustainability outcomes, while Schlosberg & Collins [24] investigate community justice’s role in environmental stewardship. These studies show that fulfilling SDG 16 requires not only the construction of fair legal systems but also a commitment to participatory governance, which ensures that justice serves as a basis for long-term societal progress.

### 2.2 Judicial system and artificial intelligence

AI holds promise in speeding up judicial adjudication by providing auxiliary help in predictive analysis, language translation, case management, e-discovery, legal research, document analysis, sentiment, audio, and video analysis. Integrating machine learning and natural language processing (NLP) has opened up numerous possibilities for significant changes in judicial adjudication [25]. Although no administrative or judicial body in the United States or other countries has implemented a system for complete decision-making through algorithms, auxiliary software like COMPAS is used for recidivism risk assessment [26]. AI systems such as ROSS Intelligence and IBM’s Watson are also being employed to analyze vast amounts of legal documents and precedents, helping legal practitioners quickly identify relevant case law and litigation strategies [6]. Similarly, Kira Systems and Luminance leverage AI to extract critical information from contracts, enhancing the speed and accuracy of contract reviews. During legal investigations, AI-powered e-discovery systems like Relativity and Brainspace efficiently sift through massive electronic document collections to locate pertinent information [27]. Businesses like LexMachina use AI algorithms to analyze historical case data, predict case outcomes, and provide attorneys with insights into legal strategies and potential risks.

Additionally, NLP tools improve legal writing by offering real-time suggestions on grammar, style, and clarity. AI-driven virtual assistants like DoNotPay help users generate legal documents, offer legal advice, and assist with basic legal procedures, making legal information more accessible. Large language models are expected to assist the judicial system further. However, while AI offers many advantages, it also presents challenges, such as bias and discrimination, and its opaqueness may impact the due process of law [28]. In the Chinese judicial system, AI has saved millions of dollars, and it could similarly reduce adjudication costs globally, potentially making the process more efficient, precise, and fair, thus increasing its legitimacy [29]. The integration of artificial intelligence in the judicial system depends on how the legislators recognize the prospect of artificial intelligence. These recognitions may lead to the proper integration of AI in the judicial system by regularizing the technology or might restrain its usage if found to be a threat to the well-being of society. These threats might include fundamental speech rights, privacy, security, and intellectual property. It is also noted that human societies have suffered in terms of social justice due to cognitive bias. Semantic bias tends to amplify cognitive bias [30]. However, leaving these concerns apart, artificial intelligence might favour the judicial system in terms of accuracy and efficiency. The above circumstantial evidence in the literature provides a basis to hypothesize that artificial intelligence can directly or indirectly affect the judicial system. Therefore, it is further hypothesized that AI and judicial systems are likely to exhibit connectivity throughout a specific time frame.

### 2.3 Judicial system and media

Media has long been a determinant in society, with its roots tracing back to cave paintings and oral storytelling. Over time, it has evolved alongside human development and societal changes [31]. The invention of the printing press in 1440 marked a significant leap, and by the 17th century, newspapers were widely regarded as reliable sources of information [32]. The 20th century saw a media explosion through radio and television. Today, in the current century, the advent of social media, as part of the fourth revolution, has connected the world, turning it into a global village. The impact of media on society, including its influence on the judicial system, is a continuous phenomenon [33]. Media can substantially influence the judicial system, mainly through investigative journalism, which holds both legal cases and the judiciary accountable [34]. However, excessive media coverage can lead to the perception of a "trial by media," which may undermine the fairness of the judicial process [35]. It can create challenges in ensuring a fair trial [36].

Public perceptions of the judiciary are also shaped by the media, as seen in a Swiss study highlighting the media’s role in agenda-setting [35]. Social media, which reaches millions globally, can quickly spread content. If that content involves defamatory statements about a judge, it could affect their selection or the fairness of a trial [37]. Media intervention may sometimes influence judicial decision-making [10]. Moreover, social media has pervasive effects across all aspects of society [38]. Both anecdotal and empirical evidence suggest that the current media revolution is already affecting the legal system [39]. These effects manifest in direct and indirect ways, shaped by various contexts. Given this evidence, it is plausible to hypothesize that media and the judicial system will continue to demonstrate increasing interconnectivity over time.

### 2.4 Judicial system and international financial institutions

There is a notable connection between the judicial system and international financial institutions (IFIs), which aim to provide development funding on a global scale [40]. These financial institutions, such as the World Bank, International Finance Corporation (IFC), International Monetary Fund (IMF), and regional development banks like the Asian Development Bank (ADB), are often supported by powerful governments that uphold the rule of law and good governance. Despite their developmental goals, IFIs can influence judicial systems by imposing conditions on governments seeking loans, sometimes proposing policies that may undermine judicial independence. The broader implications of financialization on social justice raise questions about how a deeper understanding of migrants’ experiences with financial institutions could enhance rights-based support [41]. This connection is further explored in studies differentiating between distinct "eras" of adaptation finance, particularly in the context of climate justice. A thorough analysis of justice concerns related to the evolving expectations and regulations governing adaptation funding is essential, as these issues have significant implications for climate justice [42]. Empirical evidence suggests that IFIs may impact legal systems both directly and indirectly. Hence, it is plausible to hypothesize that the relationship between IFIs and the judicial system will continue to develop and strengthen over time.

### 2.5 Judicial system and international legal influence

In the global era, the United Nations plays a crucial role in addressing emerging challenges by offering guidance to member nations, drafting conventions, and creating treaties to ensure the implementation of international legislation. To oversee compliance, the UN forms committees that act as watchdogs and may call upon the International Court of Justice to address violations by member states. While this framework can positively guide nations, it can also raise concerns about interference in local judicial systems, as each country has its legal traditions. For example, the European Court of Human Rights overruled the UK’s ban on assisted suicide [43], and international criminal courts have also been known to intervene in national decisions (Amann, 2012). International courts often become involved in human rights cases [44]. This empirical evidence leads to hypothesizing that AI, media, IFI ILI, and the judicial system might show Connectedness over a specified time frame.

### 2.6 Literature gap

A brief review of the literature highlights the significant role of artificial intelligence (AI), media, international financial institutions (IFIs), and international legal influence (ILI) in shaping judicial systems. These factors can either enhance the judicial system’s active role in society or pose risks that threaten its legitimacy. In comparison, there is evidence in the literature supporting the potential for these forces to introduce significant changes within judicial systems. This role might make the judicial system actively participate in its role in society, or these may significantly endanger its legitimacy. Their risk profile and qualities to make drastic changes within judicial systems might be optimal. A notable gap in the literature exists, as the subjectivity of AI remains unexplored, mainly in the context of judicial systems. The legal framework in pursuit of SDG-16 at the global level is missing. This study addresses this gap by examining the impact of AI, media, IFIs, and ILIs on judicial subjectivity and recommending a legal framework to mitigate these risks. By doing so, it hopes to enhance the legitimacy of the judicial system in achieving the objectives of SDG-16.

## 3. Theoretical underpinning

The judicial system is crucial in maintaining the rule of law and upholding social norms [45]. Various philosophies of justice influence judicial systems. For instance, deontological theories focus on individual rights and responsibilities, while utilitarianism aims to maximize the good for the most significant number of people. Procedural justice emphasizes fair processes, whereas distributive justice concerns itself with equitable outcomes [46]. The roots of these systems trace back to different histories; for example, the English legal system originated with the king, who was regarded as sovereign [47]. Over time, these institutions have evolved, and nations now collaborate to develop their legal frameworks [48]. An active judicial system positively impacts society and fosters social progress [49].

Currently, society is undergoing two significant transitions: a rapid population increase and technological advancements. Population growth is creating challenges, such as delays in the administration of justice and compromises in efficiency and quality. However, technological progress offers promising solutions to these issues. Artificial intelligence (AI) innovations—such as machine learning, natural language processing, and large language models—are highly beneficial in sectors including the judicial system [47]. Algorithms and large datasets are being utilized to develop models for classification and interpretation [50]. However, the opaque nature of these algorithms, combined with concerns about data quality and quantity, raises fears about their potential to disrupt established legal norms [51]. When integrated into the court system, AI may introduce bias and unfairness [52] and infringe upon privacy rights [53]. Therefore, concerns about the limitations and ethical risks of AI, such as biases that may inadvertently favor certain groups, transparency issues in algorithmic decision-making, and the potential erosion of judicial discretion are there. Additionally, international institutions like the International Monetary Fund (IMF) or World Bank, while promoting judicial reforms through funding and guidelines, may be perceived as imposing one-size-fits-all approaches that don’t always align with local legal cultures or priorities. Moreover, critics further argue that this influence may risk the sovereignty of national legal systems, pressuring them to adopt reforms that cater more to global standards than to domestic needs.

The media, often regarded as the fourth pillar of the state, could play a significant role in AI’s incorporation into the judicial system. By informing the public about the benefits of AI in legal contexts, the media could help enhance the system’s legitimacy [54]. With its dynamic platform, social media could also provide educational opportunities and facilitate discussions between AI architects and legal professionals to develop a thoughtful approach to AI integration. Additionally, the media could help ensure accountability and transparency within this process [55]. International financial institutions (IFIs) are also involved, offering support to strengthen economies, particularly during recessions [35].

Given that AI technology requires substantial data and large models for training, which demands significant financial investment, IFIs might assist technology companies in investing in AI, particularly in the judicial sector. They could finance joint ventures based on policy recommendations and help governments create regulatory frameworks for AI [56]. The influence of international law on AI integration in judicial systems cannot be overlooked. While several legislative acts, such as the AI Act in Europe, are under consideration, no unified global legal code governs AI. Countries are adopting different approaches to AI regulation. For example, the UK and the US are working towards a "self-regulation" approach [57], while Europe has passed an AI Act that imposes legal restrictions [58]. China has also introduced laws governing generative models and algorithmic ethics [59]. The fragmented global approach, exacerbated by economic, cultural, and strategic differences, may affect the integration of AI into legal systems.

This ongoing debate reveals the potential dynamics of technological innovation that will be explored in this study. By investigating these aspects, new insights into the demand-side dynamics of the legal system may emerge. While much attention has been focused on AI’s broader implications, its integration into the judicial system remains underexplored. According to the 2023 Global AI Index by Tortoise Media, AI innovations will profoundly impact the economy, society, and governance [60]. Similarly, McKinsey Global Institute’s Artificial Intelligence Guide provides insights into AI applications across various systems [61], and Stanford’s 2023 AI Index Report examines foundation models’ geopolitical and environmental implications. While the potential of AI is being actively researched, its integration into the judicial system is still an extensively unexplored area. Therefore, following this discussion, the empirical model of the study can be defined as:

JSt=f(AIt,Mdt,IFIt,ILIt) (1)

**Fig 1:**
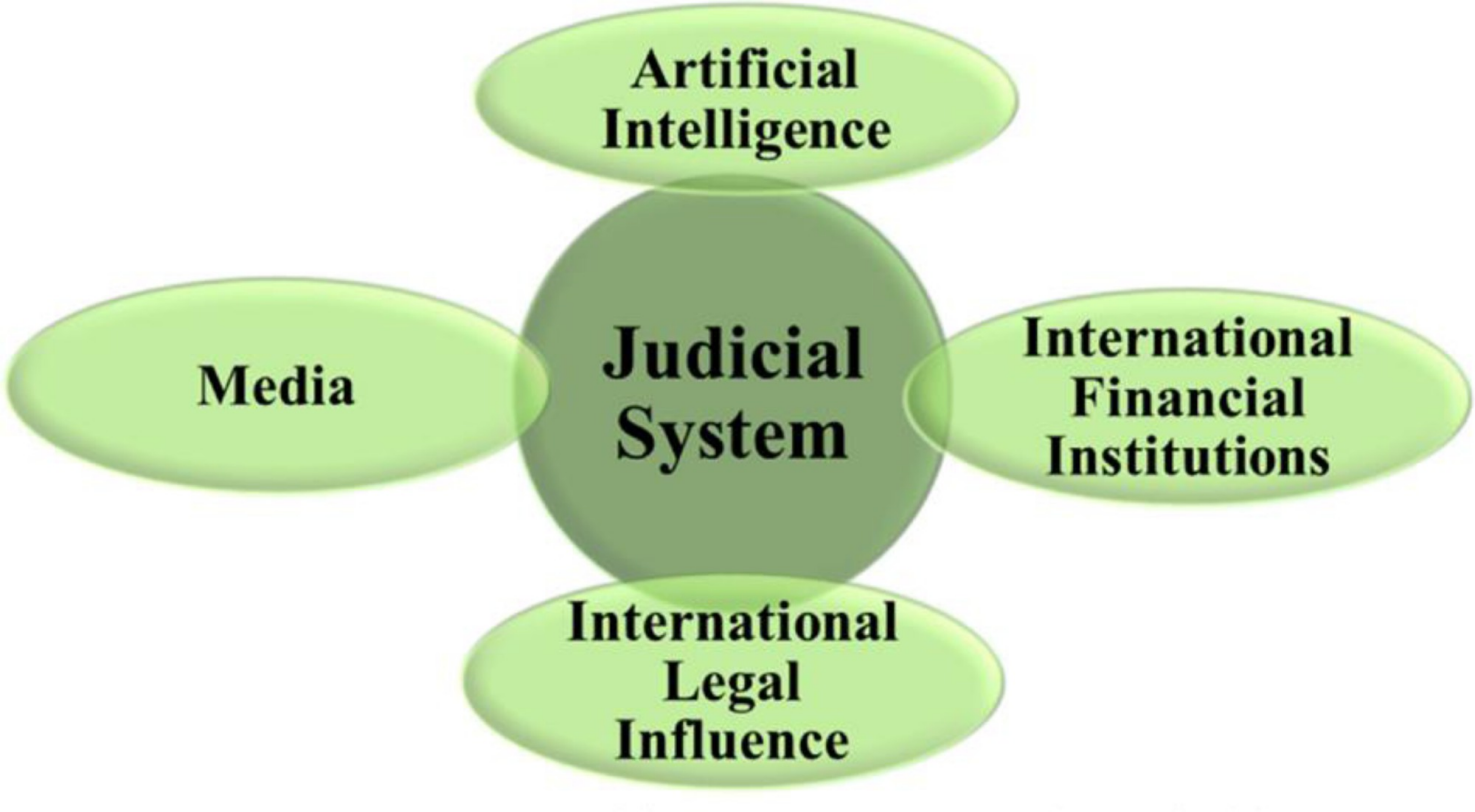
Conceptual model of factors connectedness with the judicial system. The diagram shows key factors influencing connectedness with the judicial system, including public trust, legal awareness, access to justice, and institutional transparency. Solid arrows indicate direct relationships, while dashed arrows show indirect influences. Societal factors like education and economic stability surround the core factors, highlighting their broader impact on the judicial system.

JS represents the judicial system, Md represents the media, IFI represents international financial institutions, and ILI represents international legal influence. Fig 1 denotes the connectedness of variables. The function f(.) defines the nature of the association between the driver of the judicial system and artificial intelligence. The expected impacts of the policy and environmental parameters on the energy transition can be outlined as follows:

MarginalEffectofArtificialIntelligence:ΔJSt/ΔAIt>0 (2)

MarginalEffectofMedia:ΔJSt/ΔMdt>0 (3)

MarginalEffectofInternationalFinancialInstitutions:ΔJSt/ΔIFIt>0 (4)

MarginalEffectofInternationalLegalInfluence:ΔJSt/ΔILIt>0 (5)

## 4. Data and methodology

### 4.1 Data and preliminary analysis

Examining the potential impact of artificial intelligence (AI), media (Md), international financial institutions (IFI), and international legal influence (ILI) on the judicial system (JS), the recent article employs weekly data spanning from January 13, 2019, to January 14, 2024, based on data availability. The chosen data range of 2019 to 2024 corresponds to current breakthroughs and increased implementation of AI technologies in the judicial sector, making it a timely timeframe for investigating AI’s impact on judicial justice and efficiency. This time is a transformative moment in which AI applications in law, such as predictive analytics, natural language processing for legal research, and case management systems, have acquired substantial popularity. By focusing on this recent chronology, our study represents the current and growing environment of AI within judicial processes, ensuring that the results are relevant to continuing discussions on AI’s role in justice systems. Moreover, our data is weekly data, hence providing detailed results. However, because this index is available with daily data but the other study of our series is available in weekly data so, to integrate AI data with other series, we follow and convert the daily data of the AI index into quarterly data while using the quadratic with sum method in EViews-12.

The data for the artificial intelligence index, serving as a proxy for AI, is sourced from S&P Global. Additionally, the study constructs indices for Md, IFI, ILI, and JS using Google Trends to gather relevant terms in line with methodologies proposed by Chishti et al. [62], Chishti and Ritesh [63], and Khalfaoui et al. [64]. For instance, to construct the index for Md, terms such as print, social, and electronic media are analyzed. Similarly, the index for IFI is built from terms like donors, IMF, UN Conventions, World Bank, and foreign direct investment. For the ILI index, relevant terms include international influence, international court, human rights groups, and the World Bank. The JS index uses terms related to the rule of law, compliance, adjudication, security risk, privacy risk, e-evidence, and unbiased decision-making. Certain phrases were chosen for the construction of indices for variables including public trust, judicial efficiency, and AI usage since they were pertinent to our study’s emphasis on the role of AI in judicial justice and transparency. To illustrate the range of AI’s impact on procedural justice and institutional efficacy, terminology such as "bias in AI," "algorithmic transparency," and "judicial backlog reduction" were incorporated. To make sure the index accurately reflects the main facets of AI’s judicial application, we selected phrases based on both recent policy reports and the literature. By supporting reproducibility and increasing transparency in our methods, this strategy enables future researchers to comprehend and duplicate the procedure for related studies.

**Fig 2:**
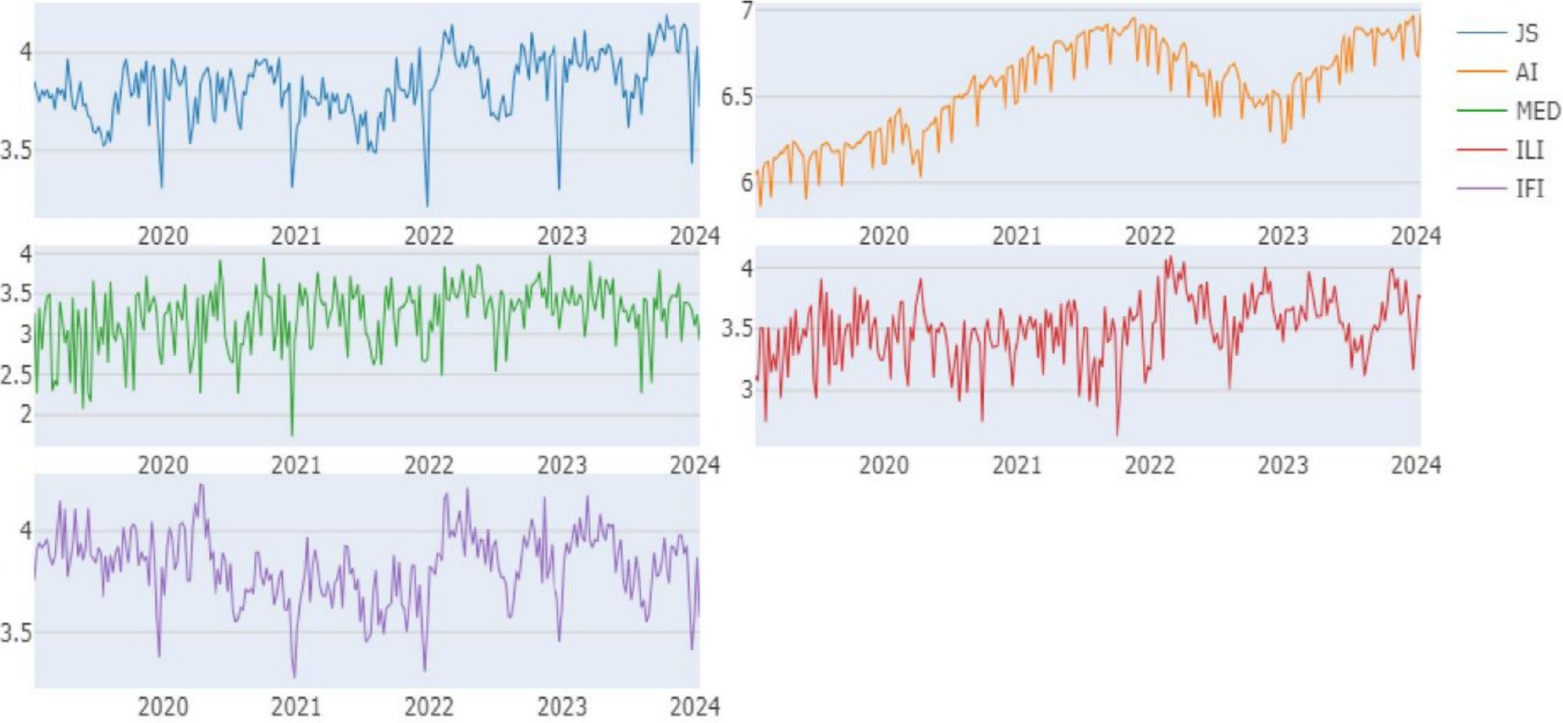
Overtrends in the modeled series. This figure illustrates the overall upward or downward trends observed in the modeled data over time, highlighting significant shifts in the pattern of the series.

Each index is computed using the arithmetic mean of the collected words, following the approaches outlined by Khalfaoui et al. [64]. All series analyzed are presented logarithmic, with Fig 2 illustrating trends in the modelled series. Table 1 provides descriptive statistics for the chosen series, offering valuable insights into their characteristics and informing the selection of appropriate empirical analysis methods. Skewness and kurtosis tests reveal that all modelled series exhibit non-normal distributions, highlighting their asymmetric nature.

**Table 1:** Descriptive statistics.

	JS	AI	MED	ILI	IFI
**Mean**	3.823	6.548	3.218	3.499	3.824
**Variance**	0.029	0.074	0.153	0.069	0.03
**Skewness**	-0.539***	-0.354**	-0.890***	-0.432***	-0.334**
	-0.001	-0.019	0	-0.005	-0.027
**Ex.Kurtosis**	0.673**	-0.944***	0.681**	0.25	0.126
	-0.047	0	-0.045	-0.326	-0.534
**JB**	17.630***	15.209***	39.657***	8.836**	5.049*
	0	0	0	-0.012	-0.08
**ERS**	-4.113***	0.344	-5.096***	-2.034**	-4.049***
	0	-0.731	0	-0.043	0
**Q(10)**	246.256***	1031.540***	34.800***	124.513***	259.136***
	0	0	0	0	0
**Q2(10)**	261.871***	1034.862***	35.555***	136.602***	261.428***
	0	0	0	0	0

Table posits different test details for JS, AI, MED, ILI and IFI whereby it is worth noting that *, **, and *** indicate the significance level at 10%, 5%, and 1%, respectively.

The results from the Jarque-Bera test further confirm this observation, emphasizing the need for nonlinear or asymmetric methods, as linear approaches may produce biased results, as noted in prior studies [65]. Following recommendations from related research, this study employs wavelet quantile correlation (WQC), a nonlinear method, to ensure the reliability and accuracy of the findings. Additionally, Table 1 presents the ERS unit root test outcomes, confirming that all series are stationary and facilitating further analysis using the WQC method. The comprehensive daily data collected over these five years is crucial for capturing the dynamics and nuances of these variables, ultimately enhancing the validity of the research findings. Moreover, the selection of the span from 2019 to 2023 is mainly due to the vast innovation of AI tools and their use in judicial adjudication. Likewise, during this span, forces like media, IFI, and ILI also seem to be more relevant.

### 4.2 Methodology

Wavelet Quantile-Co relation (WQC) approach is particularly effective in capturing the complex, time-varying influences of the independent variables on the dependent variables across the short, medium, and long run. Moreover, the WQC approach is capable of detecting potential asymmetries in the relationships between variables while presenting results across various quantiles. It also can account for the effects of outliers by segmenting outcomes into distinct quantiles. In this study, the WQC method is used to examine the correlation between artificial intelligence, media, international financial institutions, international legal institutions, and judicial systems. Unlike conventional econometric methods, the WQC approach is better suited to uncover the intricate relationships between the independent variables and judicial systems.

Kumar and Padakandla [66] implemented the Wavelet Quantile Correlation (WQC), a cutting-edge methodology. Besides, this technique is an extended description of the quantitative correlation method developed by Li et al. [67]. It was developed to investigate the correspondence amongst two (X, Y) variables, Li et al. [67]. It was put forward that Q_τ,X_ can be denoted as the τ^th^ quantile of X, and similarly Q_τ,Y_(X) can be denoted as the τ^th^ quantile of Y while X is denoted for the condition. Further, X is an independent series.

The following mathematical equation is a justification for the quantile covariance:

$${qcov}_{t}(Y,X)=cov\{I(Y-Q_{\tau ,Y}>0,x)\}=E(\varphi_{\tau }(Y-Q_{\tau ,Y})(X-E(Y))$$ (6)

Where 0<τ<1.

φ_τ_(w) = τ−I(w<0).

Finally, Li et al. (2015) finds that the QC can be calculated as:

$${qcov}_{t}(Y,X)=\frac{{qcov}_{t}(Y,X)}{\sqrt{{(var(\varphi }_{\tau }(Y-Q_{\tau ,Y})var(X)}}$$ (7)

The QC approach is modified by Kumar and Padakandla [66] in such a way that a maximum overlapping discrete wavelet transform (MODWT) developed by Perciwal and Walden [68] is utilized to decompose the X_t and Y_t. At the j^th level, the pairs of X_t and Y_t are decomposed, and then quality control procedures are performed to obtain the weighted quality control (WQC) for each level j. Therefore, Kumar and Padakandla [66] calculate the WQC by using the expression that is presented below:

$${WQC}_{\tau }(d_{j}[X],d_{j}[Y]=\frac{{qcov}_{t}(d_{j}[X],d_{j}[Y]}{\sqrt{var(\theta_{\tau }(d_{j}[Y]-Q_{\tau ,d_{j}[Y]}))var(d_{j}[X])}}$$ (8)

Where X and Y are the independent and dependent series, respectively, in Eq (8); in addition, we divided our period into three scales: the short run, the medium run, and the long run. It was done to effectively capture the more informative behaviour across both the dependent and independent series. In addition, the WQC approach can capture the possibilities of asymmetric relationships between the model parameters while simultaneously portraying the results in a variety of quantiles. Based on the findings of Chishti et al. [69], the WQC approach can also handle the effects of the outliers as shocks by dividing the outcome into quantiles.

## 5. Results

### 5.1 Full sample analysis

This subsection computes the marginal effects of independent variables on the dependent variable, the judicial system (JS). The study utilizes the wavelet quantile correlation method to analyze outcomes across different time horizons: short, medium, and long runs. The short run corresponds to 2–4 weeks, the medium run to 8–16 weeks, and the long run to 32–64 weeks. Before interpreting the results, it is crucial to understand the layout of the heatmap: the vertical axis represents the various periods, the horizontal axis represents different quantiles, and the colour bar on the right indicates whether the independent variable’s impact on the dependent variable is positive, insignificant, or negative. For this analysis, we assume that the short run captures cases of shorter duration, the medium run corresponds to cases of moderate duration, and the long run reflects the period for resolving long-duration cases. On the horizontal axis, quantiles from 0.01 to 0.3 represent lower quantiles, associated with a low frequency of cases; 0.04 to 0.6 represent medium quantiles, linked to a moderate case frequency; and 0.07 to 0.99 reflect higher quantiles, indicating a high frequency of cases being processed in courts on average.

**Fig 3:**
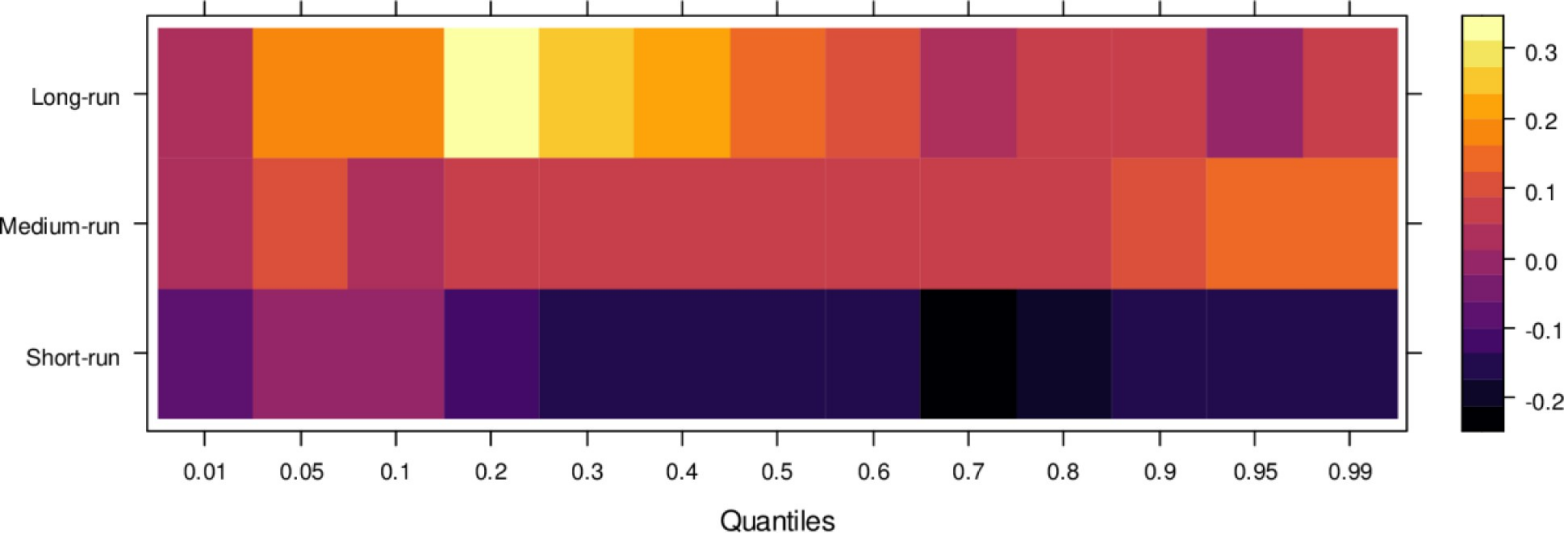
Effects of AI on the judicial system. This figure depicts the impact of AI technologies on various aspects of the judicial system, including decision-making efficiency, bias mitigation, and judicial responsiveness.

Examining the impact of AI on the judicial system (JS), as depicted in Fig 3, reveals that AI exerts adverse effects across most quantiles, from lower to higher, in the short run. It suggests that AI introduces bias when used to assist the judicial system during this period. In contrast, AI’s effects are primarily insignificant during the medium run, except for a few higher quantiles, where a positive impact is observed. It implies that while JS does not benefit significantly from AI in handling cases accurately in the medium run, there is some improvement in the limited range of higher quantiles as AI’s influence shifts in the long run, providing substantial benefits to JS in lower to medium quantiles by enabling accurate case resolutions with reduced bias. However, AI continues introducing bias in higher quantiles as case frequency increases. In summary, AI presents challenges in maintaining impartiality in court cases across various quantiles, particularly in the short and long runs. However, it shows promise for assisting in fair case conclusions over time.

**Fig 4:**
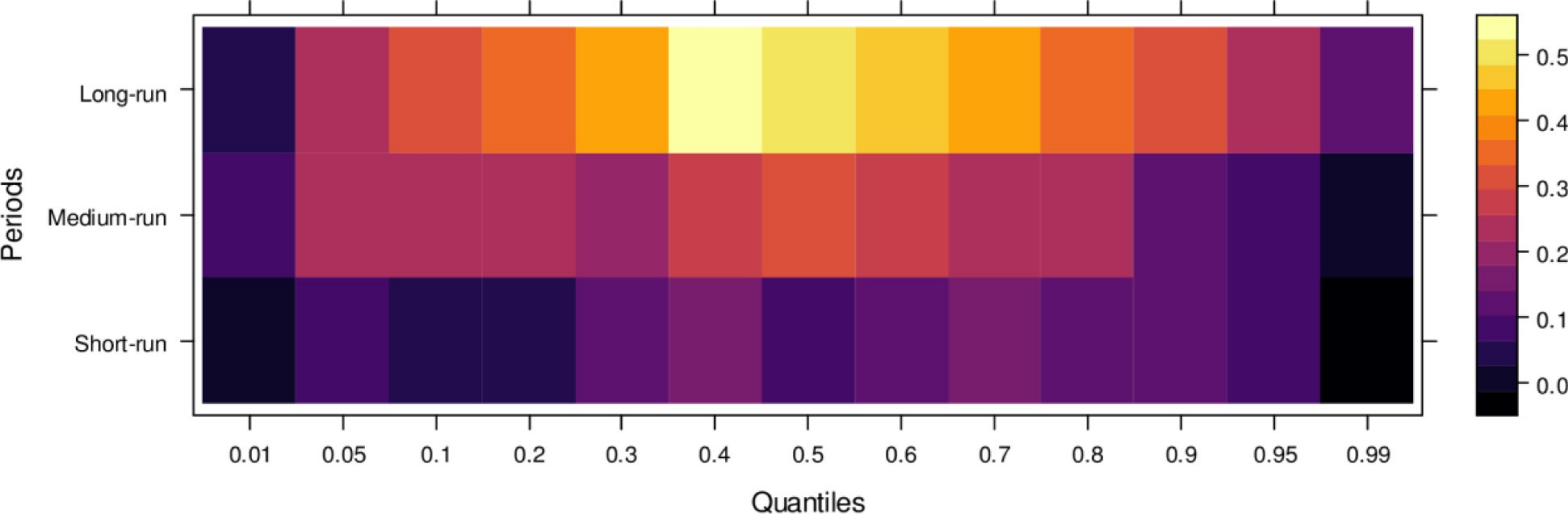
Effects of media on the judicial system. This figure illustrates the influence of media coverage on public perception, judicial decision-making, and the overall integrity of the judicial process.

Similarly, Fig 4 illustrates the impact of media on JS, showing that media has an insignificant effect on JS in the short run across lower to higher quantiles, indicating little to no influence. However, in the medium run, media demonstrates positive effects on JS across most quantiles, except for a few higher quantiles, where the impact remains insignificant. It suggests that, in the medium run, media contributes positively to JS’s ability to handle cases accurately, though with some limitations. Over the long run, media’s influence becomes increasingly positive across various quantiles, promoting accurate case resolutions and minimizing bias. However, there remain some quantiles where the effect is less pronounced. In conclusion, while the media’s short-run impact is negligible, it can play a vital role in supporting the judicial system in the long run by aiding in the just resolution of cases.

**Fig 5:**
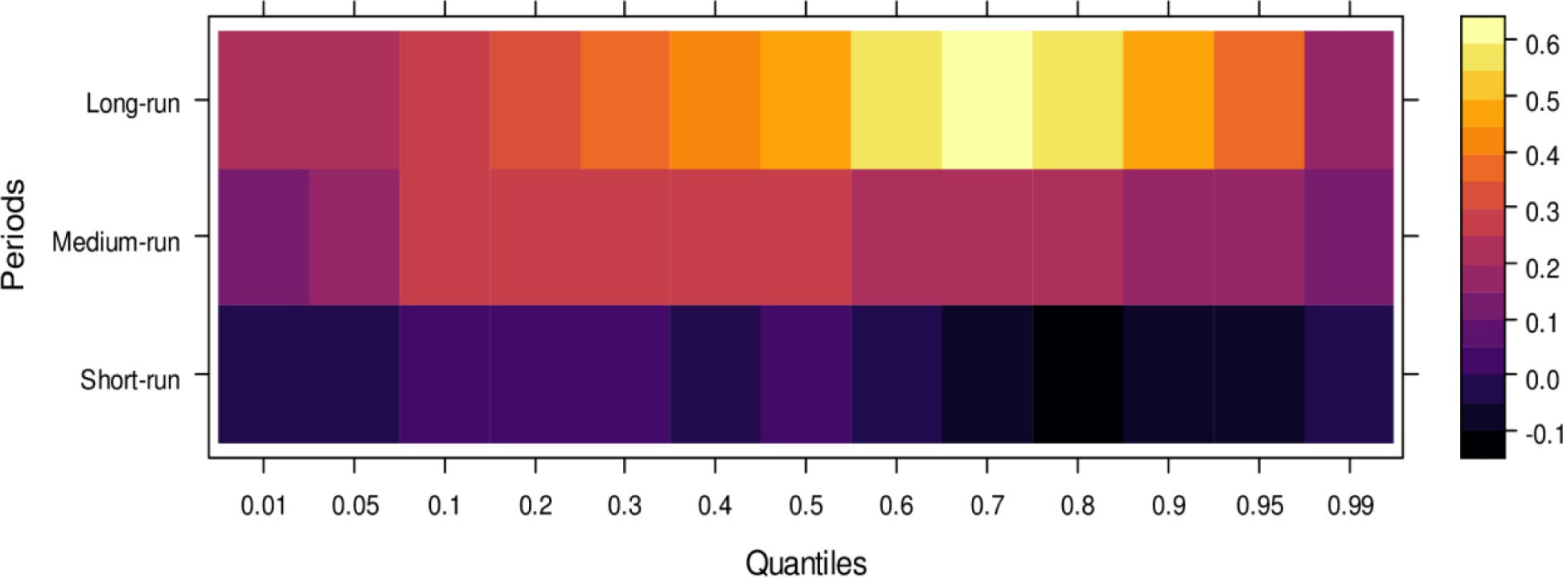
Effects of international legal influence on the judicial system. This figure shows how international legal frameworks, treaties, and foreign judicial precedents impact the decisions and practices within domestic judicial systems.

Regarding the influence of international legal standards, as shown in Fig 5, the short-run effects are largely insignificant in the lower quantiles but turn negative as they progress through the middle and higher quantiles. In the short run, it suggests that international legal influence introduces bias into JS across a broad range of quantiles. However, in the medium run, the impact shifts to significantly positive across most quantiles, with only a few higher quantiles showing an insignificant effect. It indicates that, in the medium run, JS benefits from international legal influence in handling cases more accurately. Over the long run, this positive trend persists, with international legal influence significantly aiding JS in resolving cases fairly across lower to higher quantiles, with only minor variations in the results. In summary, while international legal influence introduces bias in the short run, it becomes a valuable asset to JS in the medium and long runs by facilitating more precise and unbiased case conclusions.

**Fig 6:**
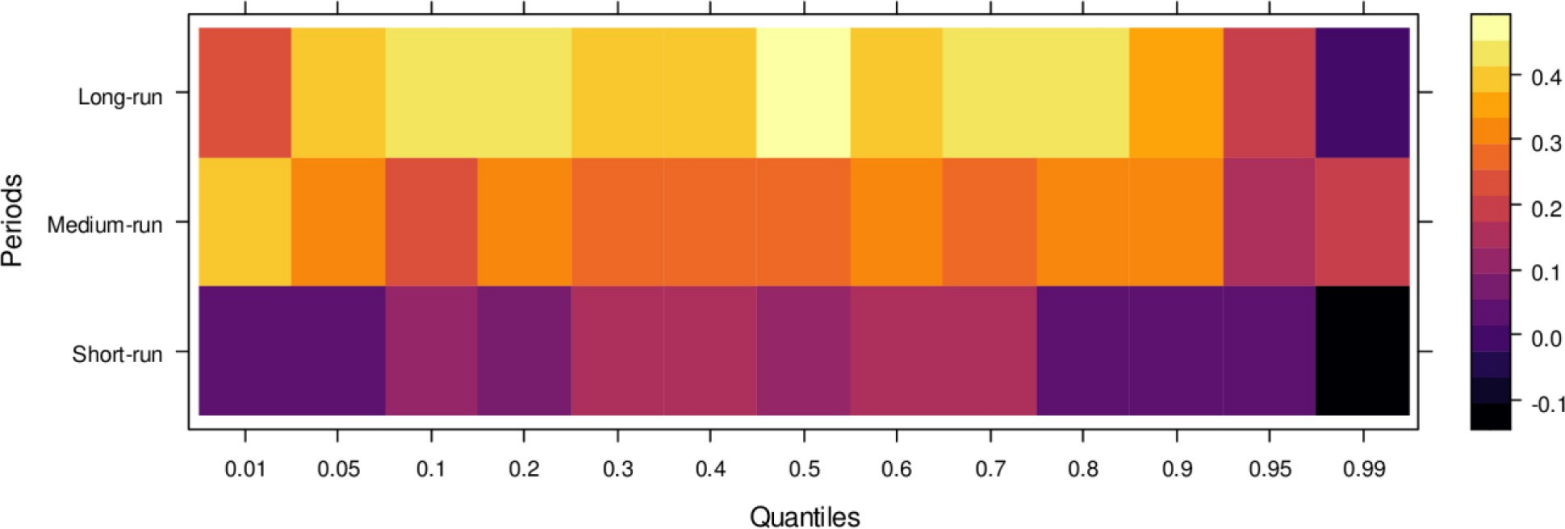
Effects of international financial institutions on the judicial system. This figure illustrates the influence of international financial institutions, such as the IMF and World Bank, on the judicial system, particularly in terms of economic policy, legal reforms, and judicial independence.

Finally, Fig 6 highlights the impact of international financial institutions on JS. In the short run, the effects are mixed, with some lower quantiles showing insignificant or positive impacts while higher quantiles reveal an insignificant or negative influence. This indicates that international financial institutions can. In the medium run, however, their impact is predominantly positive across most quantiles, with only a few higher quantiles showing an insignificant effect, suggesting substantial assistance to JS in handling cases accurately. Over the long run, the positive influence of international financial institutions becomes more pronounced, aiding in unbiased case conclusions across lower to higher quantiles, with few exceptions. In summary, although international financial institutions may introduce bias in the short run, their long-term potential to support JS in achieving precise and fair case outcomes is significant.

### 5.2 Heterogeneous analysis

This subsection calculates the marginal effects of independent variables on the heterogeneous dependent variable, namely the judicial system (JS). The study employs the wavelet quantile correlation method, which provides outcomes for various time horizons, including short, medium, and long runs. Before delving into the interpretation of results, it is essential to note that 1–2 weeks represent the short run, 4–8 weeks represent the medium run, and 16–32 weeks represent the long run. Additionally, it is worth noting that the left vertical axis of each heatmap represents the various periods, the horizontal axis represents the various quantiles, and the colour bar along the right side of each heatmap assists in understanding whether the influence of the independent variable on the dependent variable is positive, insignificant, or negative. We also assume that the short run demonstrates a period in which short-duration cases are handled, the medium run represents a period for medium-duration cases, and the long run signifies a period in which long-duration cases are resolved. On the horizontal axis, 0.01 to 0.3 quantiles represent the lower quantiles with a low frequency of cases, 0.04 to 0.6 quantiles represent the medium quantiles with a moderate frequency of cases, and 0.07 to 0.99 quantiles represent the higher quantiles with a high frequency of cases in the courts, on average.

#### 5.2.1 Relationship during 2019

**Fig 7:**
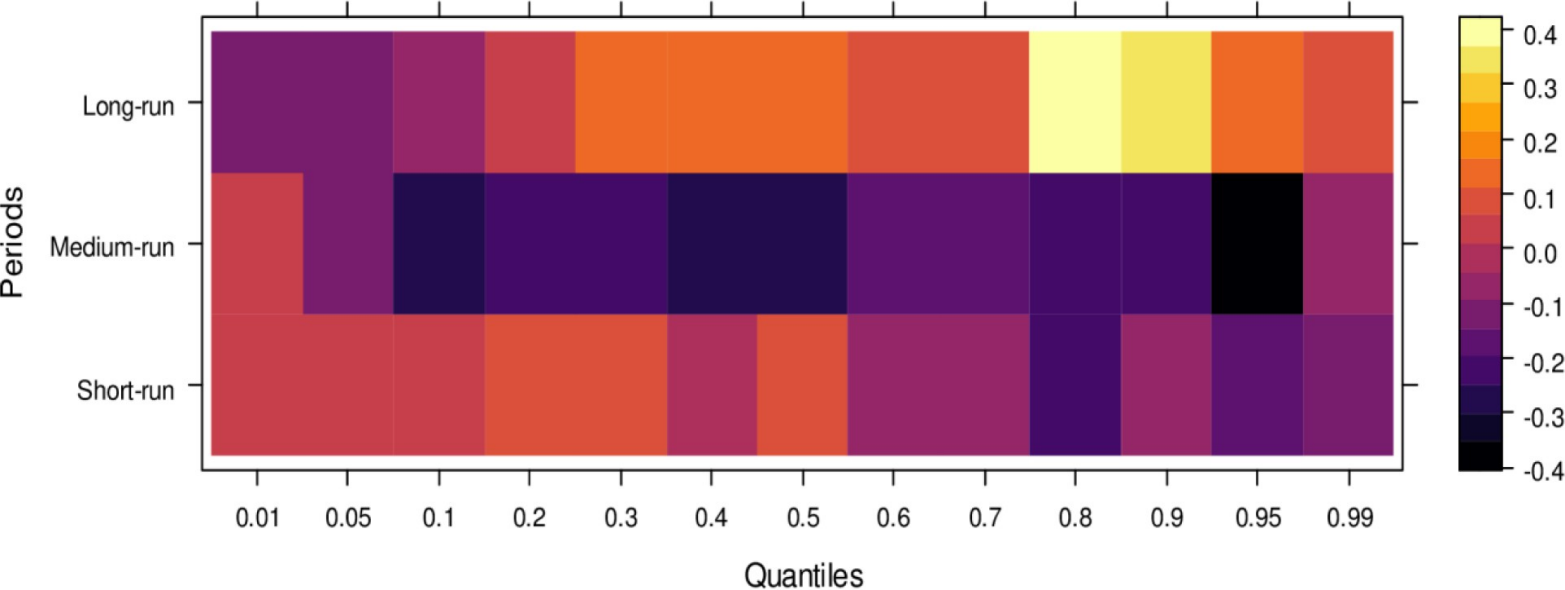
Effects of AI on the judicial system. This figure shows the impact of AI on judicial efficiency, decision-making, and bias reduction during 2019, while also addressing challenges related to fairness and accountability in the legal process.

Examining the impact of AI on the judicial system (JS) as depicted in Fig 7, it becomes clear that in the short run, AI has mixed effects across lower quantiles, with both significant and insignificant outcomes extending into the medium quantiles. However, in the higher quantiles, AI shows predominantly negative effects, reflecting a combination of adverse and insignificant impacts on JS. It suggests that AI has the potential to introduce bias when used to assist the judicial system. AI’s influence remains largely negative across most quantiles in the medium run, though there are a few insignificant effects in both the lower and higher quantiles. It indicates that AI does not provide substantial positive assistance in handling court cases during this period, except for these limited quantiles.

**Fig 8:**
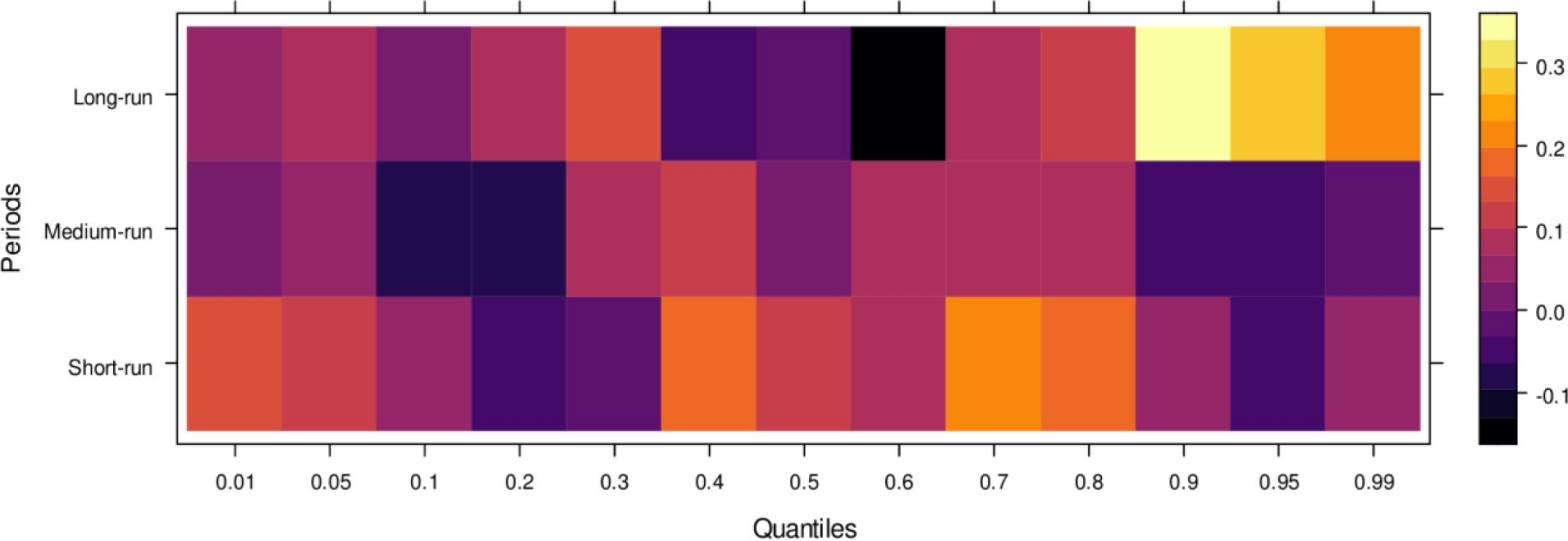
Effects of media on the judicial system. This figure illustrates the influence of media on judicial processes during 2019, including public perception, case outcomes, and the potential for bias in legal proceedings.

Nevertheless, AI’s long-term effects reveal a more positive trend. JS benefits from AI in lower to medium quantiles, allowing for accurate case conclusions without bias, mainly when case numbers are lower to medium. Higher quantiles also show significant positive effects in the long run, suggesting that AI reduces bias as case frequency increases. While AI introduces bias across medium-run quantiles, its long-term potential to assist in accurate, unbiased case resolutions is evident. Regarding media’s impact on JS, as illustrated in Fig 8, the short-run effects are similarly mixed across all quantiles, implying that media has a limited tendency to introduce bias when assisting the judicial system. In the medium run, media’s impact is primarily insignificant in the lower and higher quantiles, while medium quantiles show a positive effect, resulting in an overall mixed influence. It suggests that media does not strongly influence JS in accurately handling court cases during the medium run. However, the long-run effects show a more nuanced picture. While media has no significant impact in the lower quantiles, it turns negative in some medium quantiles but becomes positive in higher quantiles. It indicates that, in the long run, JS stands to benefit from media’s influence in lower to medium quantiles, especially when case frequency is lower to medium. In summary, although media may introduce bias across most quantiles from the short to the long run, its long-term potential to assist in accurate case conclusions, particularly in higher quantiles, is notable.

**Fig 9:**
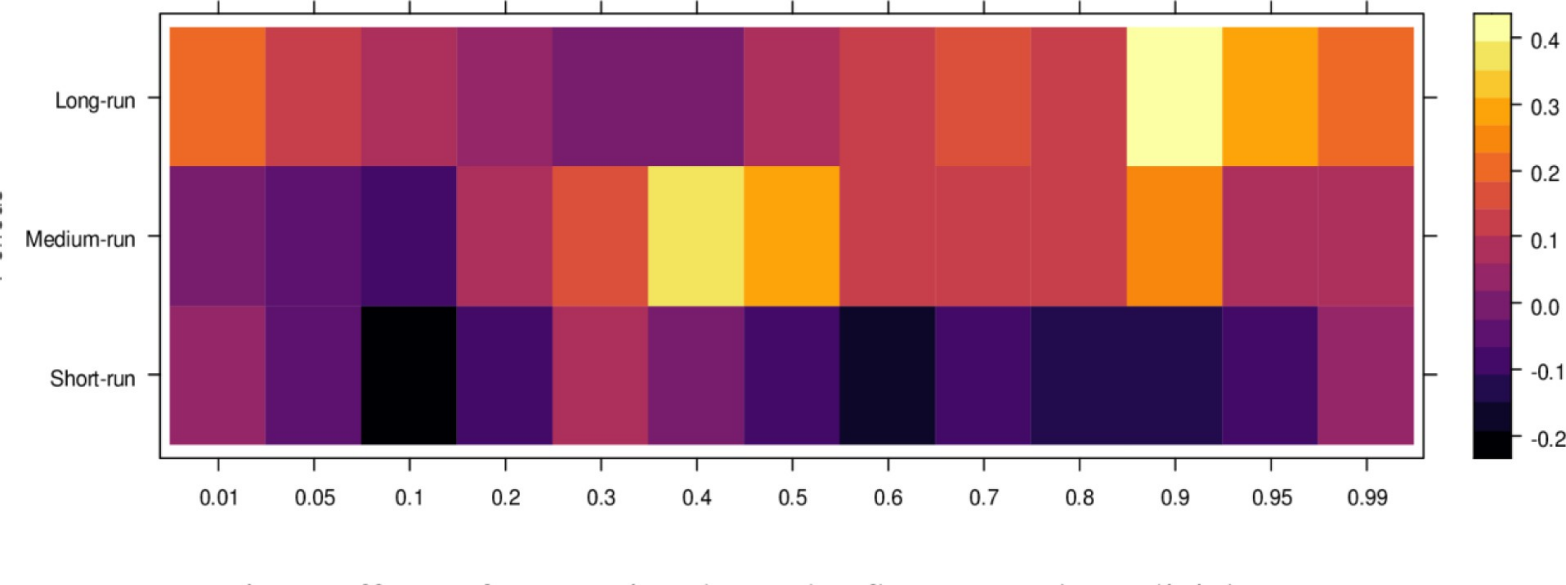
Effects of international legal influence on the judicial system. This figure depicts the impact of international legal norms and practices on domestic judicial systems during 2019, including the adoption of global standards and cross-border legal cooperation.

Probing the impact of international legal influence on JS, as seen in Fig 9, reveals that this influence has adverse effects across most quantiles, from lower to higher, in the short run. It implies that international legal influence tends to introduce bias when assisting the judicial system during this period. In the medium run, the effects remain negative in the lower quantiles but shift to positive in the medium to higher quantiles. It indicates that, in the medium run, JS receives substantial positive assistance from international legal influence in handling court cases accurately, except for a few lower quantiles. In the long run, the positive impact of international legal influence becomes more pronounced across lower and higher quantiles, allowing JS to conclude cases accurately without bias, particularly when case frequency is either low or high. However, some medium quantiles exhibit adverse effects, suggesting that bias may still occur when case frequency is moderate. In summary, while international legal influence introduces bias in the short run, its medium- and long-run potential to assist courts in reducing bias and accurately concluding cases is significant.

**Fig 10:**
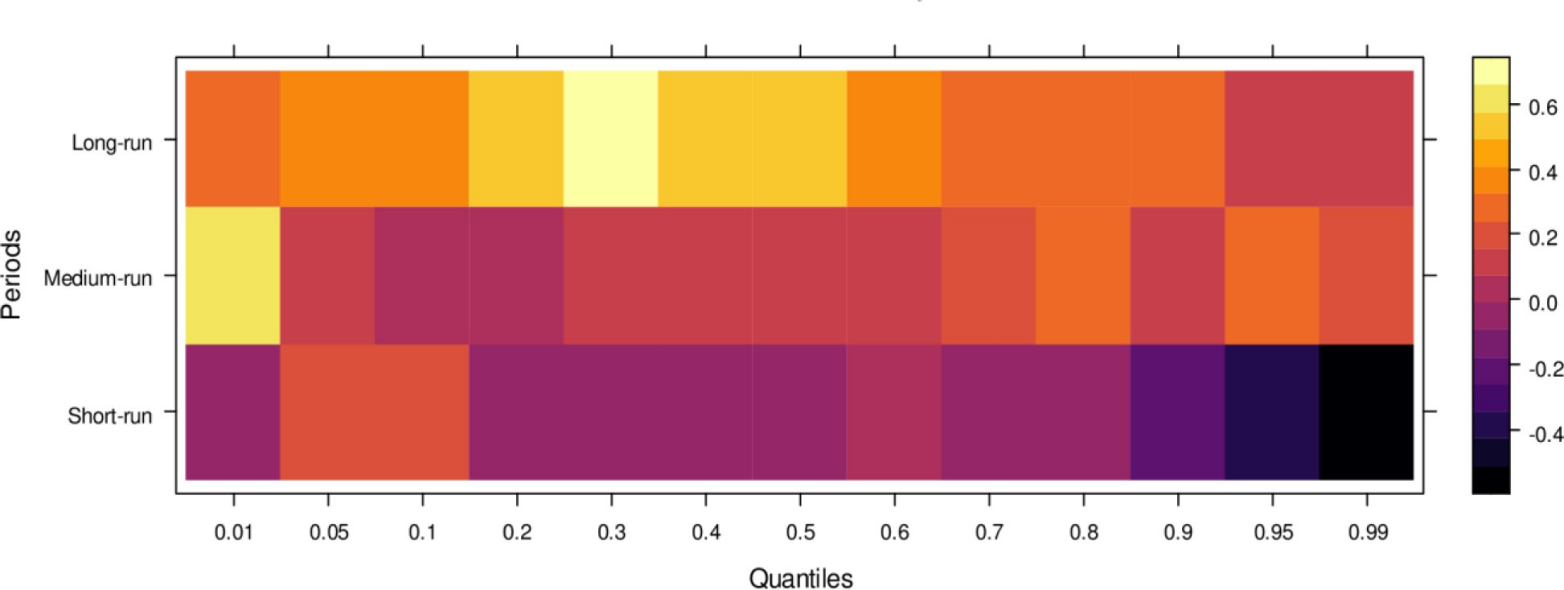
Effects of international financial institutions on the judicial system. This figure highlights the influence of international financial institutions on judicial reforms during 2019, including their role in shaping legal frameworks, ensuring accountability, and promoting economic stability through legal structures.

Finally, inspecting the impact of international financial institutions on JS, as depicted in Fig 10, reveals that their overall effects on JS are mixed in the short run. Some lower quantiles show positive results initially, but this shifts to insignificant and then adverse effects in the medium quantiles. In contrast, the medium and long runs depict predominantly positive outcomes, highlighting the potential of international financial institutions to assist JS in the long run. In summary, although international financial institutions may introduce bias in the short run, they demonstrate strong potential to aid the judicial system in accurately concluding cases and minimizing bias over the medium and long term.

#### 5.2.2 Relationship during the year 2020

**Fig 11:**
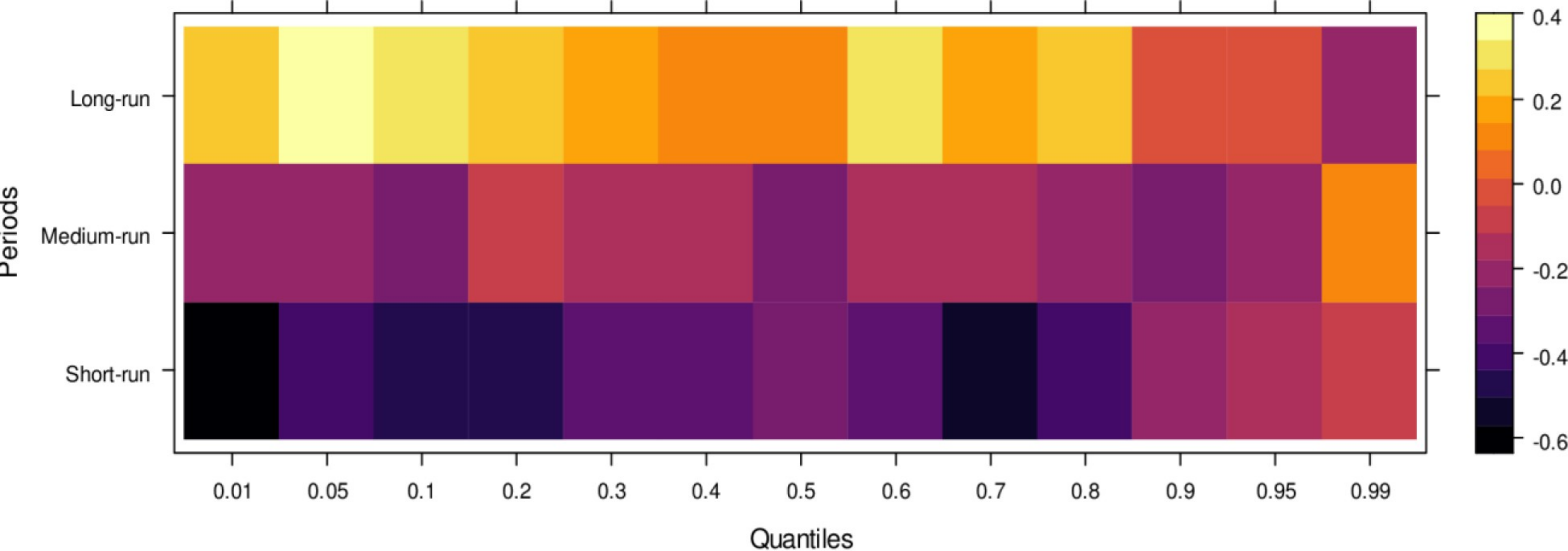
Effects of AI on the judicial system. This figure illustrates the role of AI in the judicial system during 2020, focusing on its impact on case processing, decision-making efficiency, and the management of legal resources.

Examining the impact of AI on the judicial system (JS) as depicted in Fig 11, it is clear that in the short run, AI has predominantly adverse effects across the lower quantiles, with similar results extending to the medium quantiles. In contrast, the higher quantiles show insignificant impacts, indicating a mixture of adverse and neutral effects on JS. It suggests that AI introduces bias when employed to assist the judicial system. AI continues to show negative effects across most quantiles in the medium run, with only a few higher and lower quantiles reflecting insignificant results. It implies that AI does not provide substantial positive assistance in handling court cases accurately during the medium run, except for a limited range of quantiles. However, AI’s influence shifts in the long run, showing significant positive effects across lower to high quantiles, allowing JS to resolve cases more accurately without bias, regardless of case frequency. In the long run, the higher quantiles demonstrate a particularly positive influence of AI, suggesting a reduction in bias as the frequency of cases increases. In summary, although AI introduces bias in court cases across short and medium-run quantiles, it can assist courts in accurately concluding cases and minimizing bias in the long run.

**Fig 12:**
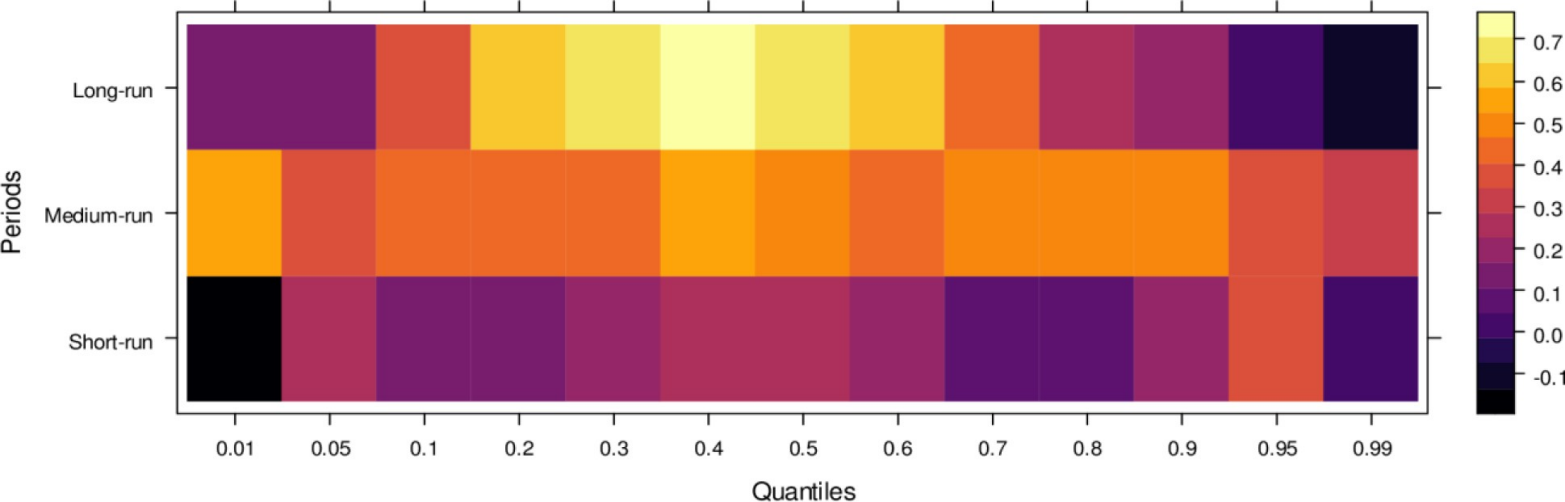
Effects of media on the judicial system. This figure shows the influence of media on the judicial system in 2020, highlighting its impact on public opinion, legal outcomes, and the potential for media-driven bias in court proceedings.

Exploring the impact of media on JS, as depicted in Fig 12, reveals mixed effects across most quantiles in the short run. It suggests that media, like AI, may introduce some tendencies toward bias when assisting the judicial system. In the medium run, however, media shows positive effects across lower and higher quantiles, indicating that it can help JS during this period. Conversely, the long-run results are mixed: the lower quantiles show adverse effects, the middle quantiles are insignificant, and the higher quantiles again turn negative. This pattern suggests that media influences JS in cases with both low and high frequencies while having little to no effect on cases of medium frequency. In the long run, media offers significant benefits in medium quantiles, where the frequency of cases is moderate, by supporting accurate, unbiased conclusions. In summary, while media may introduce bias in some quantiles from the short to long run, it can potentially assist courts in reaching fair conclusions, especially in the medium run.

**Fig 13:**
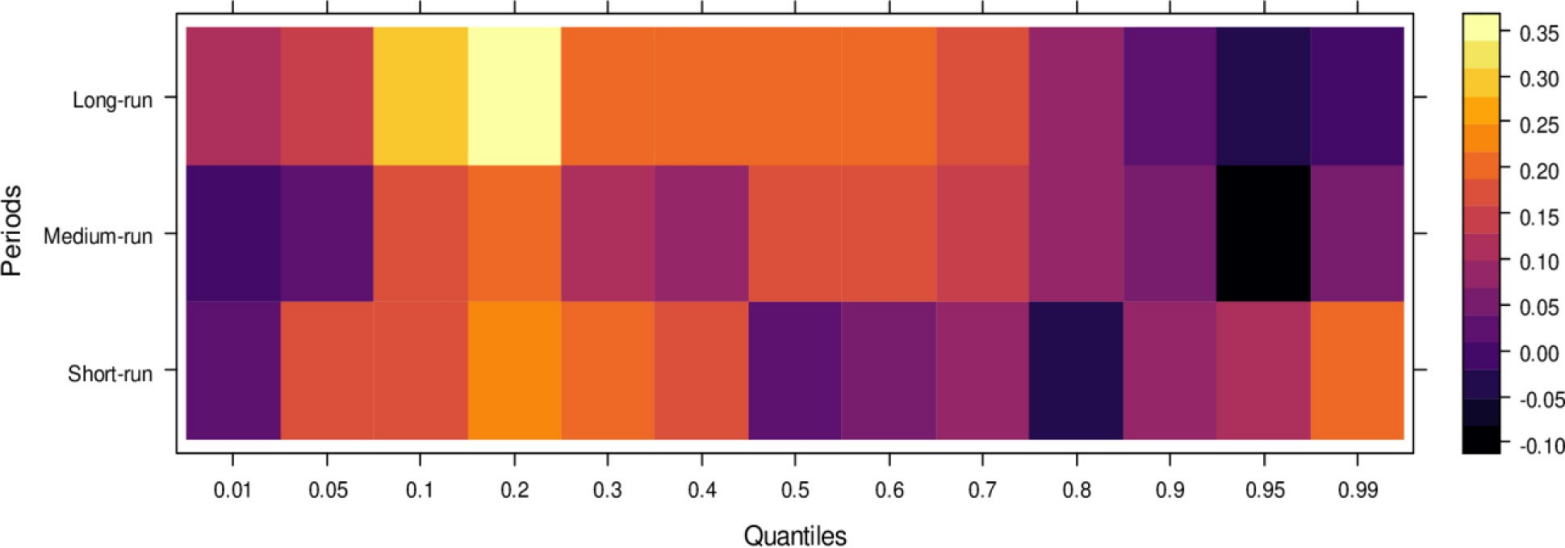
Effects of international legal influence on the judicial system. This figure illustrates the impact of international legal standards and practices on domestic judicial systems in 2020, focusing on the adoption of global norms and cross-border legal collaborations.

Investigating the impact of international legal influence on JS, as depicted in Fig 13, shows that the results are generally positive in the short run, though some quantiles exhibit insignificant effects. The results are primarily insignificant in the medium quantiles, while higher quantiles display a mix of negative and positive outcomes. These mixed results suggest that international legal influence can have both positive and negative impacts on JS, with a tendency to introduce bias when employed in the short run. In the medium run, international legal influence shows mixed results across the quantiles, indicating that JS receives varied assistance in handling cases. However, in the long run, JS benefits significantly from AI’s assistance across lower and higher quantiles, allowing for accurate, unbiased case resolutions when case frequency is either low or high. On the other hand, some higher quantiles demonstrate a negative influence of international legal influence, suggesting that bias may still arise in cases with higher frequency. In summary, while international legal influence introduces bias in court cases from the short to long run, there is potential for it to assist courts in reducing bias and concluding cases accurately over time.

**Fig 14:**
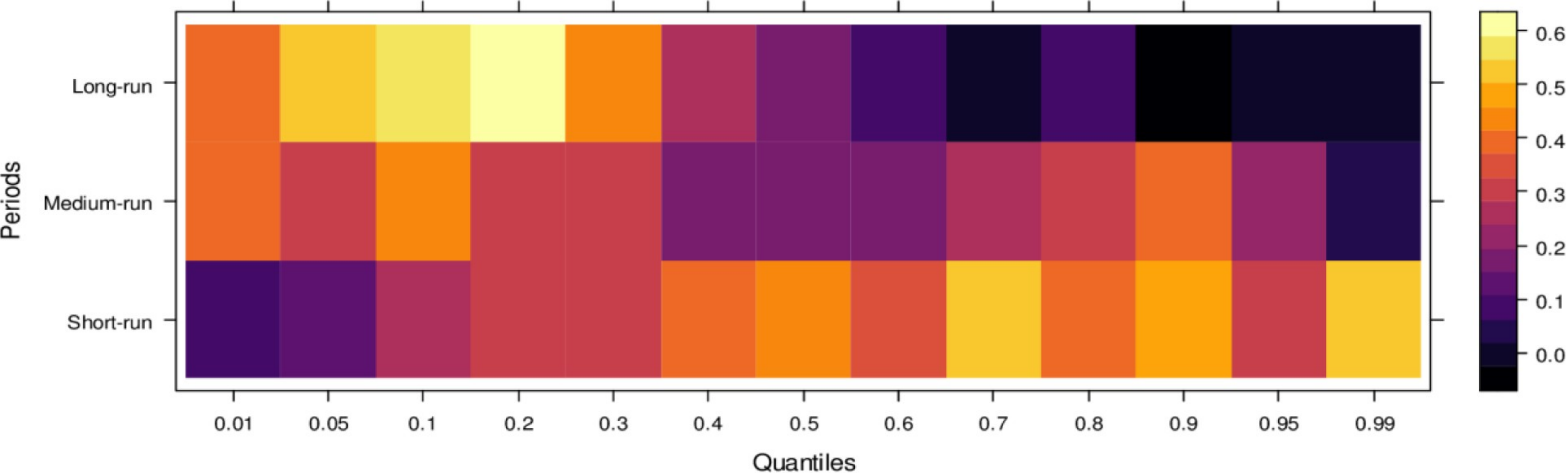
Effects of international financial institutions on the judicial system. This figure depicts the influence of international financial institutions on judicial reforms in 2020, highlighting their role in shaping legal frameworks and promoting economic stability through legal systems.

Finally, examining the impact of international financial institutions on JS, as depicted in Fig 14, it becomes apparent that these institutions have an overall positive effect on JS. In the short run, some lower quantiles show insignificant results, while medium and long-term quantiles exhibit predominantly positive outcomes, indicating that international financial institutions are generally helpful to JS. In the medium run, lower quantiles show positive effects, while medium quantiles reflect a mix of insignificant and positive results. However, this trend reverses in the higher quantiles, with positive results shifting to negative, a pattern replicating in the long run across lower to higher quantiles. In summary, while international financial institutions reduce bias in most court cases from the short to long run, there is a risk of bias being introduced in cases with high frequency in the long run. Nonetheless, these institutions show strong potential for assisting JS in handling cases accurately and minimizing bias across a broad range of quantiles.

#### 5.2.3 Relationship during the year 2021

**Fig 15:**
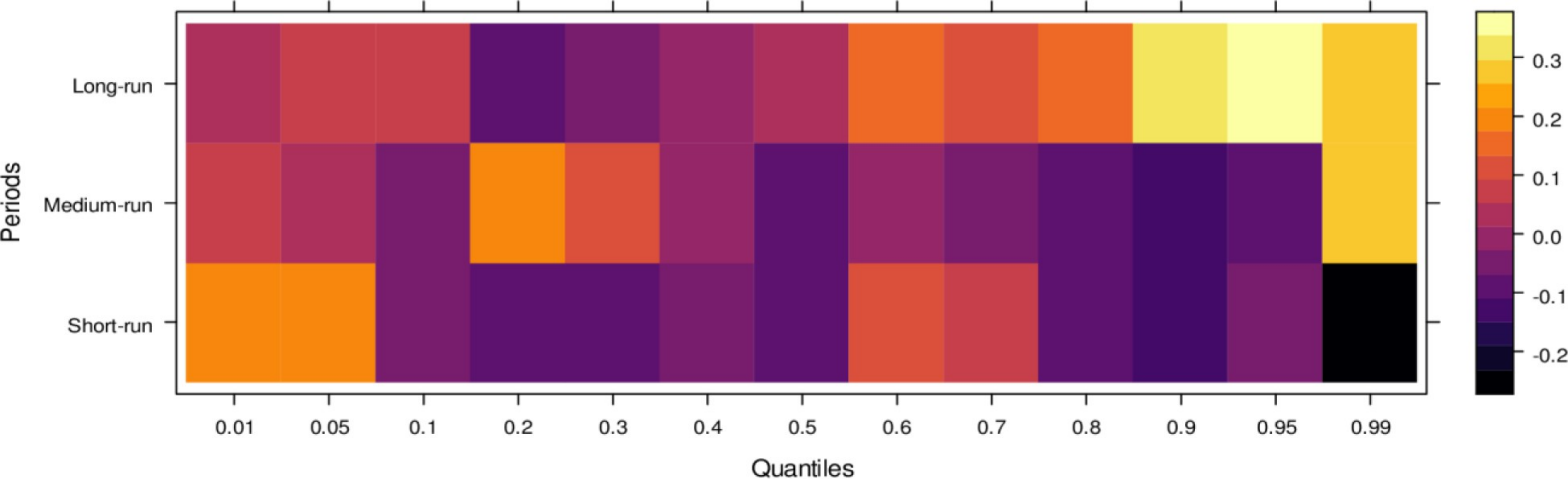
Effects of AI on the judicial system. This figure illustrates the evolving role of AI in the judicial system in 2021, focusing on its impact on legal decision-making, case management, and efforts to improve fairness and efficiency in the court process.

Examining the impact of AI on the judicial system (JS), as depicted in Fig 15, reveals that in the short run, AI produces mixed results, with positive and insignificant effects in the lower quantiles. In contrast, the medium quantiles reflect similar patterns. In contrast, the higher quantiles show a negative impact, indicating a combination of adverse and insignificant effects on JS. It suggests that AI has the potential to introduce bias when used to assist the judicial system. In the medium run, AI generally positively impacts JS across most quantiles, though a few higher and lower quantiles continue to exhibit insignificant effects. It implies that JS benefits from AI to some extent, but the assistance is limited to specific quantiles. Over the long run, AI significantly benefits lower quantiles by helping JS conclude cases accurately and without bias. While medium quantiles show mixed results, with some insignificant and adverse effects, higher quantiles display a positive influence, indicating that AI helps reduce bias as case frequency increases in the long run. In summary, although AI introduces bias in some quantiles during the medium run, it shows strong potential, in the long run, to support courts in accurately resolving cases and minimizing bias, with only minor exceptions.

**Fig 16:**
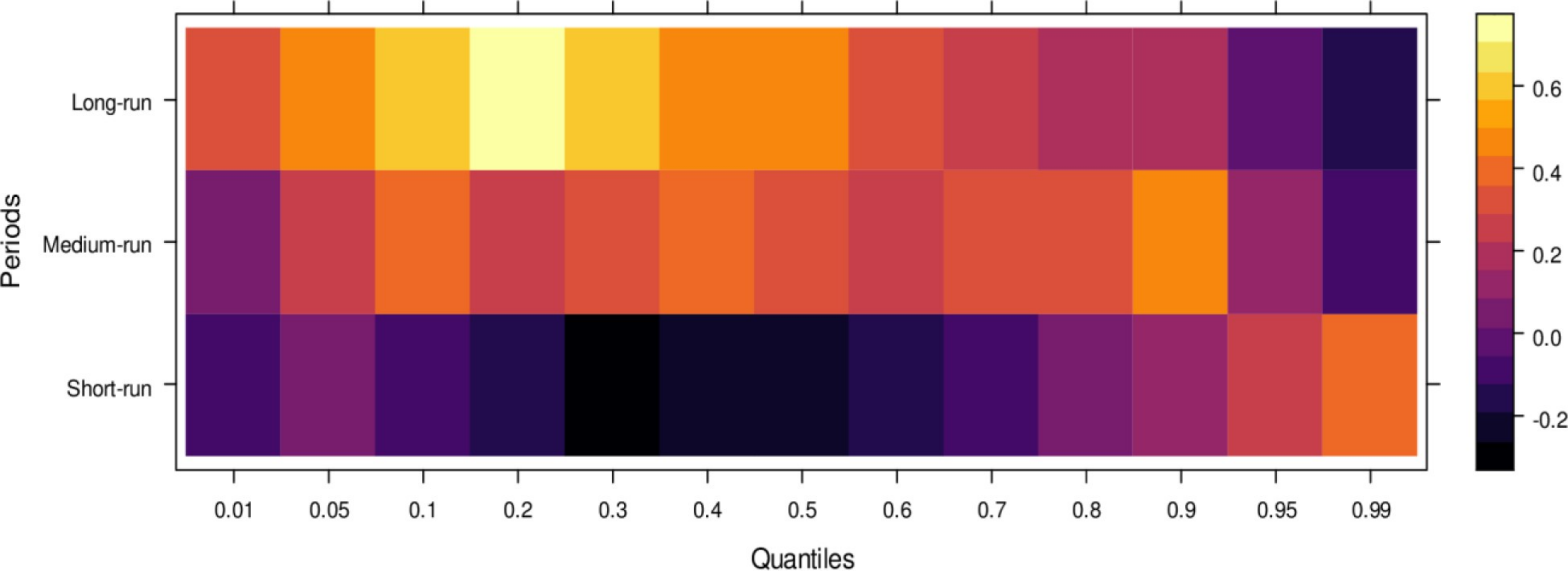
Effects of media on the judicial system. This figure highlights the impact of media on the judicial system in 2021, examining its influence on public perceptions of justice, media coverage of high-profile cases, and potential effects on legal outcomes.

Investigating the impact of media on JS, as depicted in Fig 16, shows that in the short run, media has mixed effects: insignificant in the lower quantiles, negative in the medium quantiles, and positive in the higher quantiles. It suggests that media, like AI, may introduce some bias when assisting JS in the short term. However, media produces positive results in both the medium and long runs, with only a few exceptions in the lower and higher quantiles. It indicates that JS will benefit significantly from the media’s influence, allowing for accurate and unbiased case conclusions across medium- to long-term quantiles. In summary, while media may introduce some bias in specific quantiles, especially in the short run, it holds considerable potential to support courts in achieving fair outcomes over the long term.

**Fig 17:**
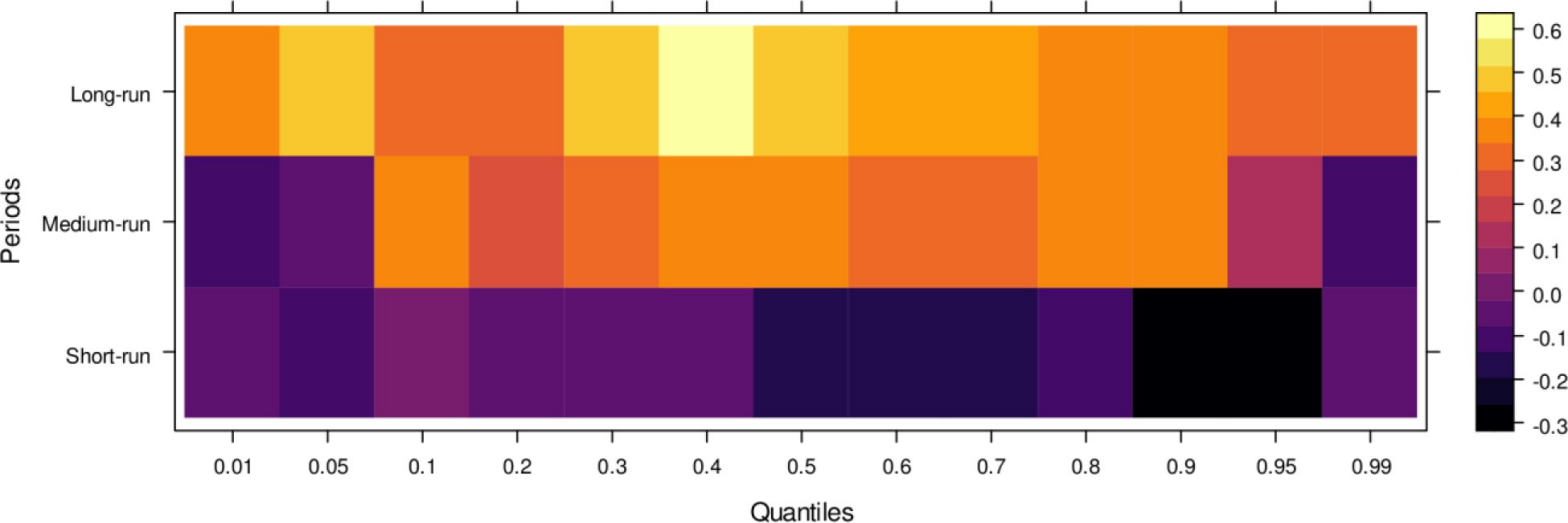
Effects of international legal influence on the judicial system. This figure shows the influence of international legal frameworks on domestic judicial systems in 2021, highlighting the integration of global legal standards and cross-border legal cooperation.

Probing the impact of international legal influence on JS, as depicted in Fig 17, reveals that in the short run, most quantiles show insignificant effects, with only a few higher quantiles indicating negative results. It suggests that international legal influence does not strongly impact JS during the short run, though there are tendencies for bias to emerge. In the medium run, international legal influence remains insignificant in the lower quantiles, but the medium quantiles show a positive impact, while higher quantiles revert to insignificance. It indicates that JS receives some positive support from international legal influence in resolving cases accurately in the medium run, though this assistance is limited to specific quantiles. In the long run, however, the influence of international legal standards becomes consistently positive across lower to higher quantiles, suggesting that JS benefits from this influence in achieving unbiased and accurate case conclusions, regardless of case frequency. In summary, while international legal influence may introduce bias in the short run, it shows significant potential in the medium to long term to assist courts in reducing bias and delivering fair case outcomes.

**Fig 18:**
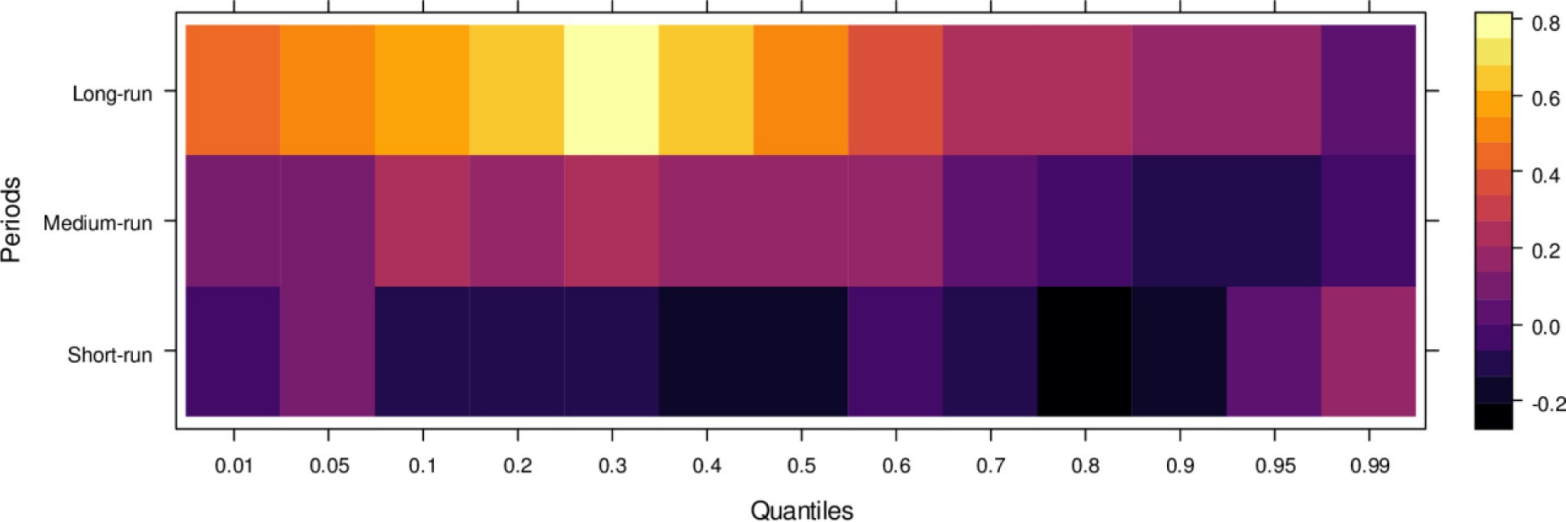
Effects of international financial institution on the judicial system. This figure shows the influence of international financial institutions on domestic judicial systems in 2021, highlighting the integration of global legal standards and cross-border financial cooperation.

Finally, examining the impact of international financial institutions on JS, as depicted in Fig 18, shows that in the short run, their effects are generally adverse, with lower quantiles reflecting insignificant results and medium quantiles transitioning from insignificant to negative. This pattern continues in the medium run, where the effects remain insignificant across most quantiles, indicating that international financial institutions do not offer substantial assistance to JS during this period. However, in the long run, lower to medium quantiles begin to show positive results, while higher quantiles remain insignificant. It suggests that while international financial institutions may not be effective in the short and medium runs, they offer potential benefits in the long run, helping courts conclude cases more accurately and with less bias. In summary, although these institutions may introduce bias in earlier phases, they demonstrate strong potential to assist the judicial system in the long run, particularly in lower to medium quantiles.

#### 5.2.4 Relationship during the year 2022

**Fig 19:**
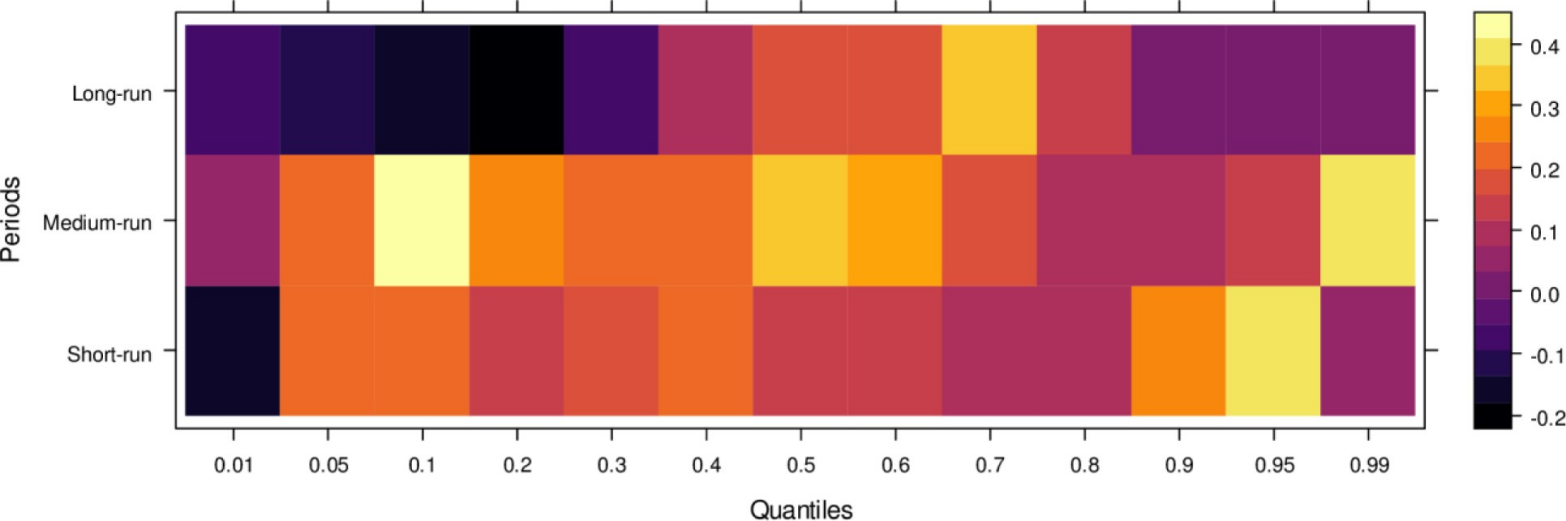
Effects of AI on the judicial system. This figure illustrates the continued impact of AI on the judicial system in 2022, focusing on advancements in legal automation, data-driven decision-making, and efforts to enhance efficiency and reduce biases in the courtroom.

Examining the impact of AI on the judicial system (JS), as illustrated in Fig 19, reveals that in the short run, the initial lower quantile shows an adverse effect, but this quickly shifts to positive across the remaining lower to higher quantiles. It suggests that AI generally has a positive impact in the short run, helping to reduce bias within the judicial system. Similarly, in the medium run, the positive trend continues consistently across all quantiles, indicating that AI supports accurate case conclusions during this period. However, in the long run, AI’s impact varies: lower quantiles shift from insignificant to negative results, representing an adverse effect, while medium quantiles remain positive, and higher quantiles turn insignificant. It indicates that AI is less effective in cases with higher frequencies in the long run but provides positive assistance in medium-frequency cases. In summary, although AI introduces some bias in the lower quantiles in the long run, it demonstrates significant potential in assisting courts in accurately concluding cases across short- and medium-run quantiles and medium quantiles in the long run, with only a few exceptions.

**Fig 20:**
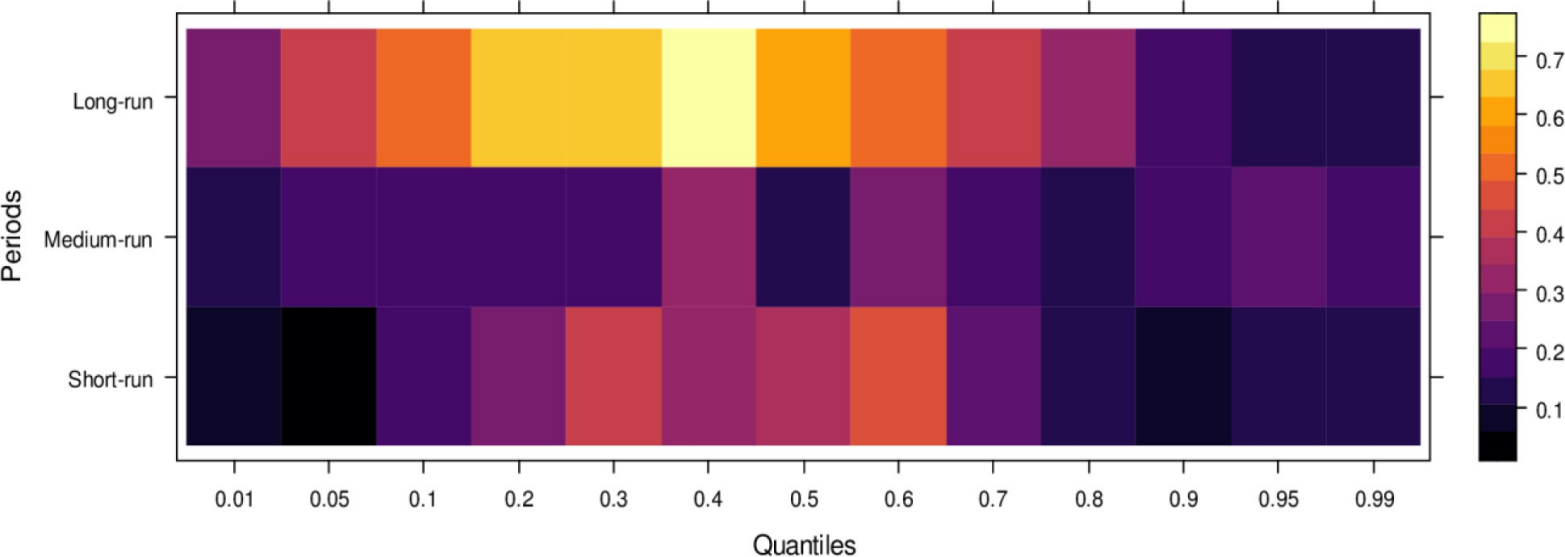
Effects of media on the judicial system. This figure shows the impact of media on the judicial system in 2022, examining its role in shaping public opinion, influencing legal outcomes, and the increasing concern over media bias in high-profile cases.

Investigating the impact of media on JS, as depicted in Fig 20, shows a uniformly positive influence across all quantiles in the short, medium, and long run. There are no negative or zero values, indicating that the media consistently supports the judicial system, helping to reduce bias and improve accuracy. Moreover, the positive impact appears to increase from the short to medium run across all quantiles. In summary, media demonstrates strong potential across all timeframes to assist courts in accurately concluding cases while limiting bias.

**Fig 21:**
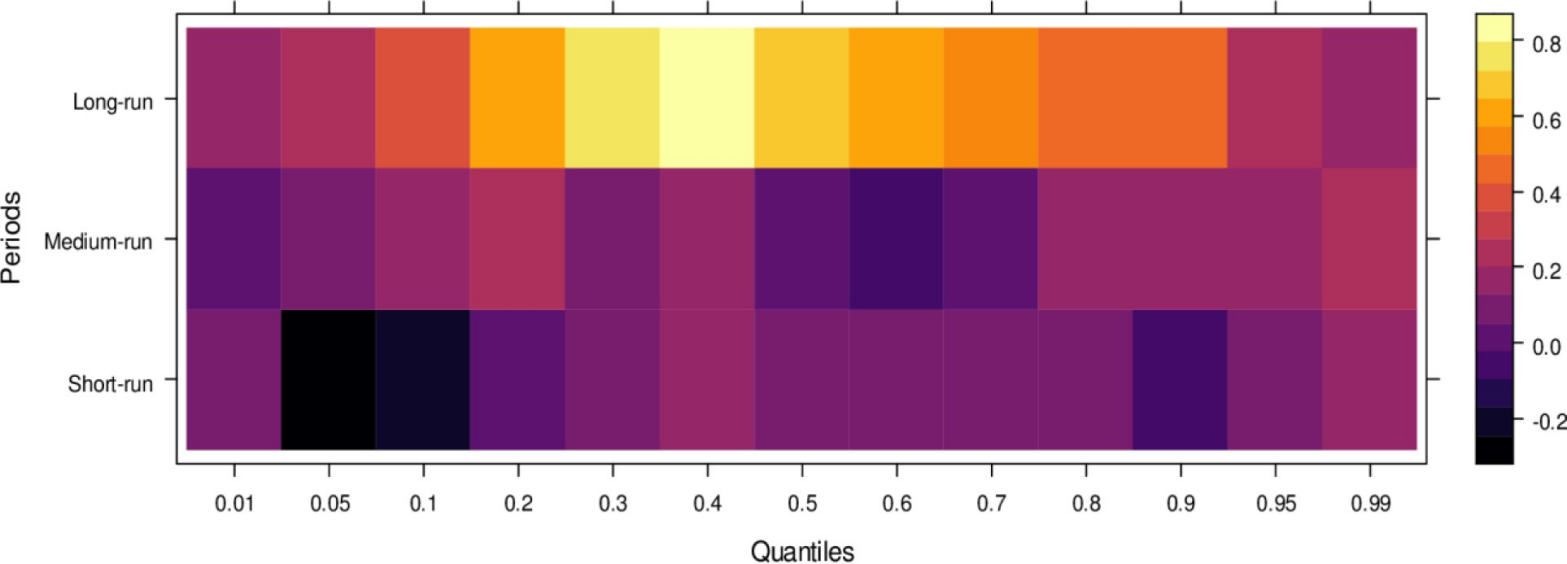
Effects of international legal influence on the judicial system. This figure illustrates the influence of international legal norms and practices on domestic judicial systems in 2022, focusing on the adoption of global standards and the strengthening of cross-border legal cooperation.

Analyzing the impact of international legal influence on JS, as shown in Fig 21, reveals a mixed impact in the short run. Lower quantiles display both insignificant and negative results, while medium quantiles remain insignificant, and higher quantiles turn positive. It suggests that the effect of international legal influence varies in the short run, increasing and reducing bias depending on case frequency. However, in the medium and long run, all quantiles’ influence becomes consistently positive, indicating a constructive effect on JS over time. In summary, although international legal influence may introduce bias in specific quantiles during the short run, it has strong potential to assist courts in accurately concluding cases and reducing bias in the medium and long run.

**Fig 22:**
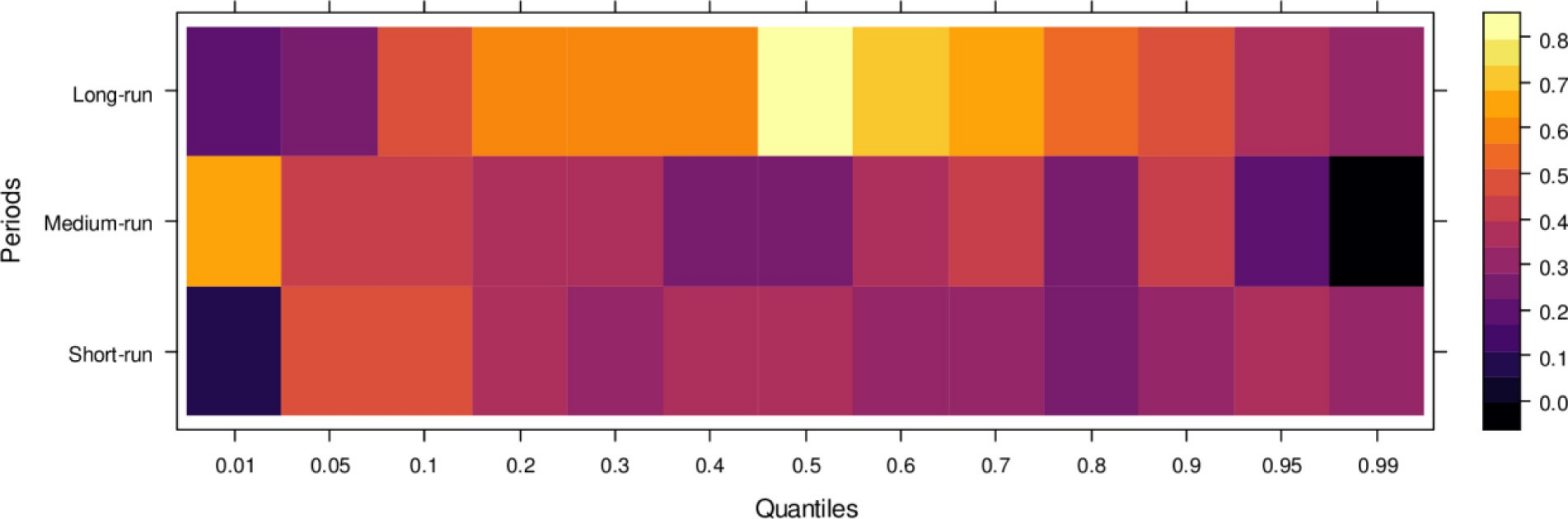
Effects of international financial institutions on the judicial system. This figure depicts the impact of international financial institutions on judicial systems in 2022, highlighting their role in shaping legal reforms, promoting financial accountability, and supporting economic stability through legal structures.

Examining the impact of international financial institutions on JS, as depicted in Fig 22, shows overall positive effects in the short run, with some lower quantiles displaying insignificant results. However, as we move to the medium quantiles, the results shift from insignificant to positive. This positive trend continues from lower to higher quantiles in the medium run, indicating that international financial institutions substantially support JS during this period. Lower to medium quantiles remain positive in the long run, while higher quantiles show insignificant effects. In summary, international financial institutions introduce minimal bias in the short run and have strong potential to assist courts in accurately concluding cases in both the medium and long run, with only a few exceptional cases of bias or insignificance in specific quantiles.

#### 5.2.5 Relationship during the year 2023

**Fig 23:**
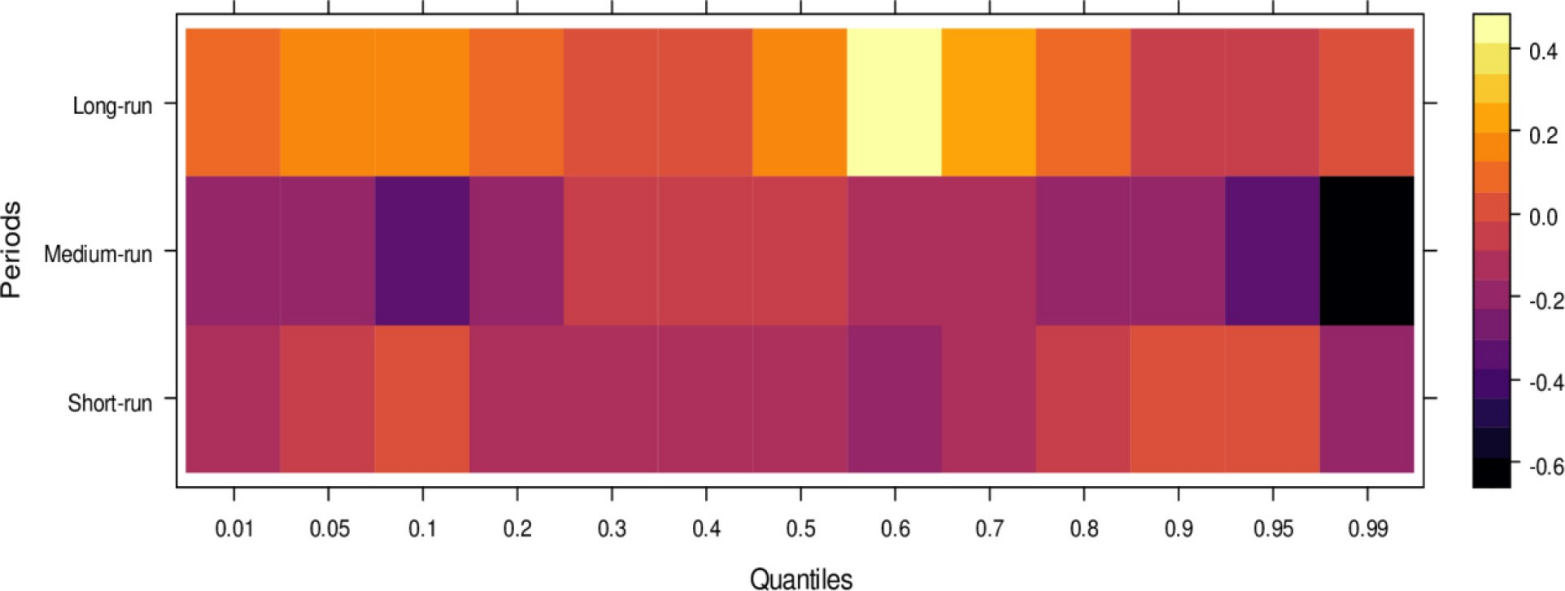
Effects of AI on the judicial system. This figure illustrates the growing influence of AI on the judicial system in 2023, focusing on innovations in legal research, predictive analytics, and the role of AI in improving case outcomes and court efficiency.

Analyzing the impact of artificial intelligence (AI) on the judicial system (JS), as depicted in Fig 23, reveals a nuanced influence across different quantiles. Lower and higher quantiles show insignificant effects in the short term, while medium quantiles indicate a negative impact. This mixed behaviour suggests that AI might introduce bias in adjudication, especially in scenarios where cases are frequent. Conversely, in the medium term, the effects vary: low quantiles indicate a negative impact, medium quantiles are insignificant, and high quantiles are negatively impacted again, highlighting an overall adverse effect or insignificance in some scenarios. In contrast, long-term results from lower to higher quantiles turn positive, suggesting AI’s potential to aid the JS in reducing bias and improving accuracy in case conclusions.

**Fig 24:**
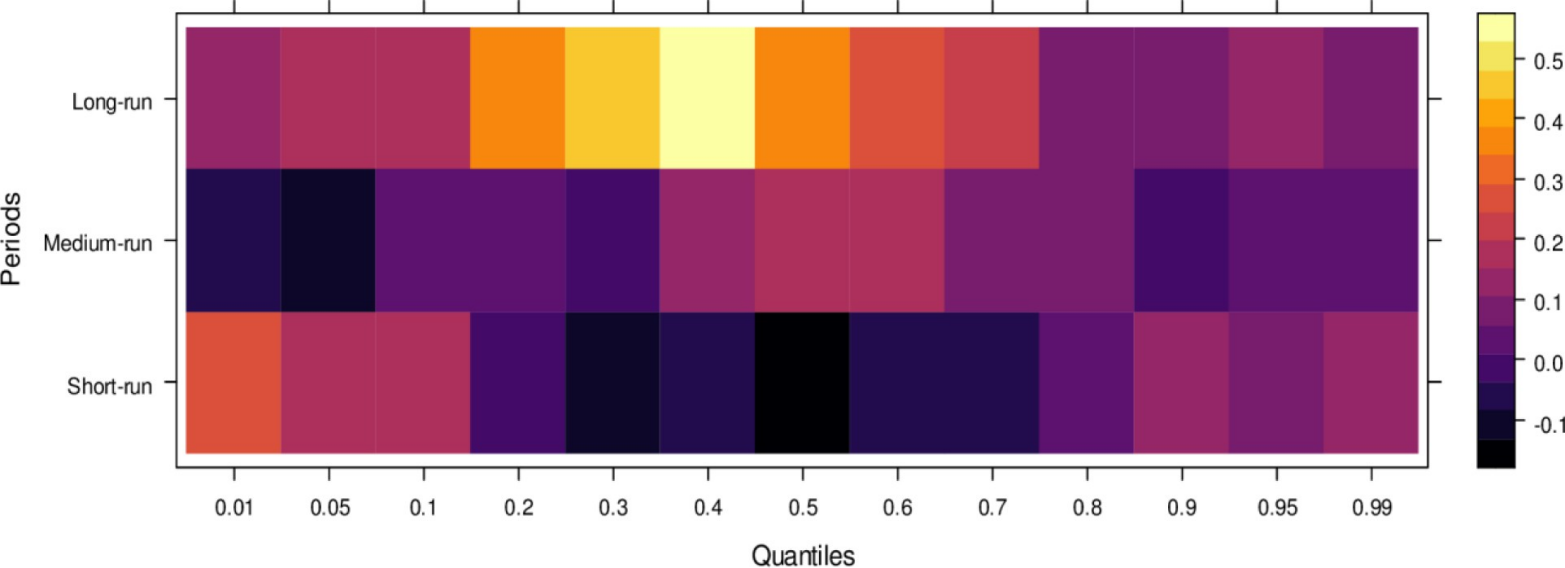
Effects of media on the judicial system. This figure highlights the continued impact of media on the judicial system in 2023, examining its role in shaping public perceptions of legal proceedings, influencing court decisions, and raising concerns about media-driven bias in high-profile cases.

Similarly, the impact of media on the JS, illustrated in Fig 24, varies over time. In the short run, lower quantiles present a mixture of upbeat, insignificant, and negative results, with medium quantiles consistently showing negative impacts and higher quantiles being insignificant. This pattern indicates an overall adverse impact of media in the medium run across all quantiles, albeit with some exceptions. However, in the long run, the influence becomes positive in lower and medium quantiles yet remains insignificant in higher ones. It suggests that media can be constructive in supporting judicial accuracy and limiting bias, mainly when case frequency is low.

**Fig 25:**
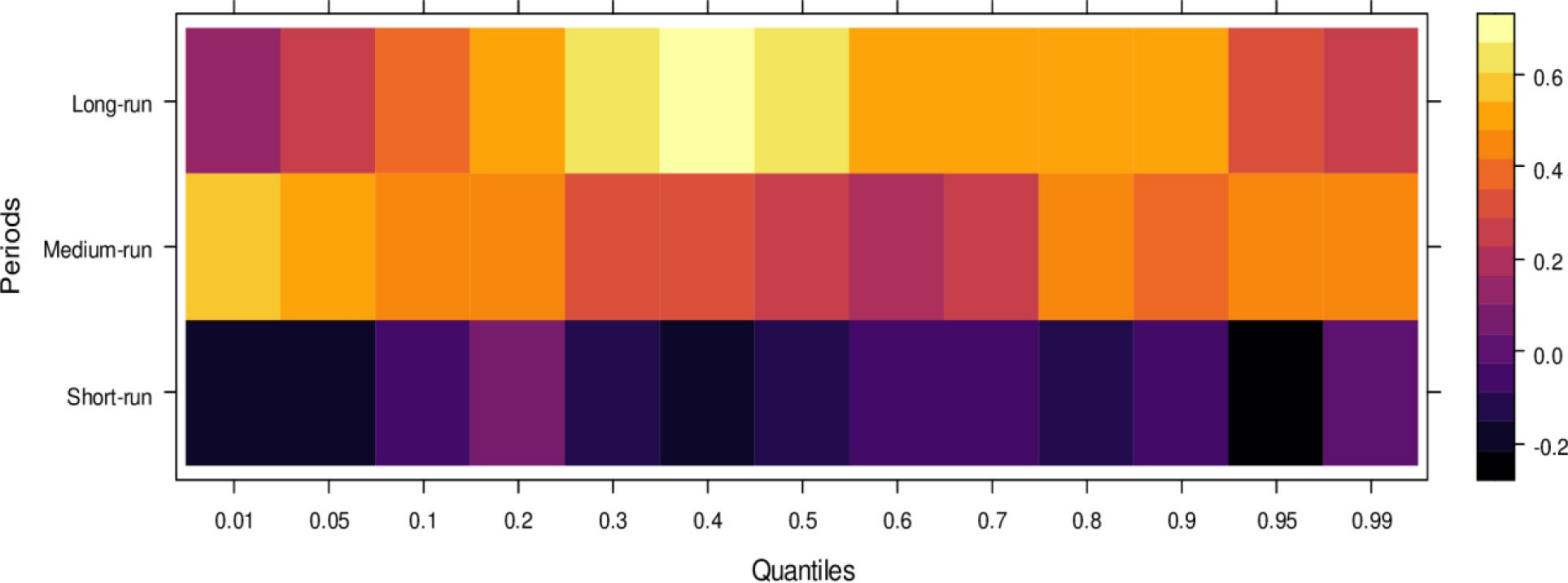
Effects of international legal influence on the judicial system. This figure illustrates the impact of international legal influence on domestic judicial systems in 2023, focusing on the adoption of global legal standards, the integration of international human rights norms, and cross-border legal collaborations.

Fig 25 analyzes the influence of international legal norms on JS, where short-run effects in lower quantiles are adverse, reflecting a tendency to introduce bias. Nonetheless, both medium- and long-run periods show positive impacts across all quantiles, indicating a strong potential for international legal norms to contribute constructively to JS, aiding in the accurate conclusion of cases and reducing bias.

**Fig 26:**
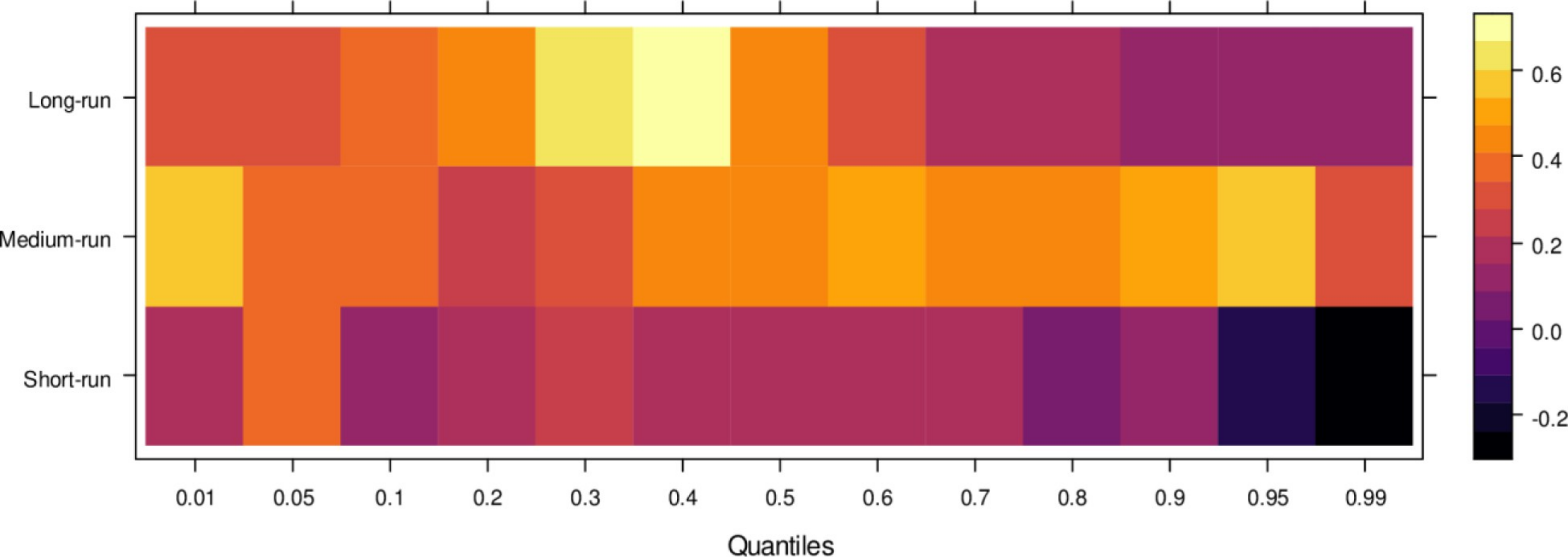
Effects of international financial institutions on the judicial system. This figure shows the influence of international financial institutions on judicial systems in 2023, highlighting their role in promoting legal reforms, supporting economic stability, and fostering financial accountability through legal frameworks.

Lastly, the role of international financial institutions, as depicted in Fig 26, exhibits generally positive short-term effects on JS across lower to medium quantiles. However, higher quantiles show some insignificant and minor negative results. In the medium and long run, the influence remains positive across all quantiles, underscoring the strong potential of these institutions to support JS by minimizing biases and enhancing the accuracy of case conclusions. In summary, while AI and media introduce biases in the short to medium term, both have the potential to support the JS positively in the long run. International legal and financial influences tend to be beneficial, particularly in longer-term evaluations, helping refine judicial processes and outcomes.

#### 5.2.6 Robustness check

**Fig 27:**
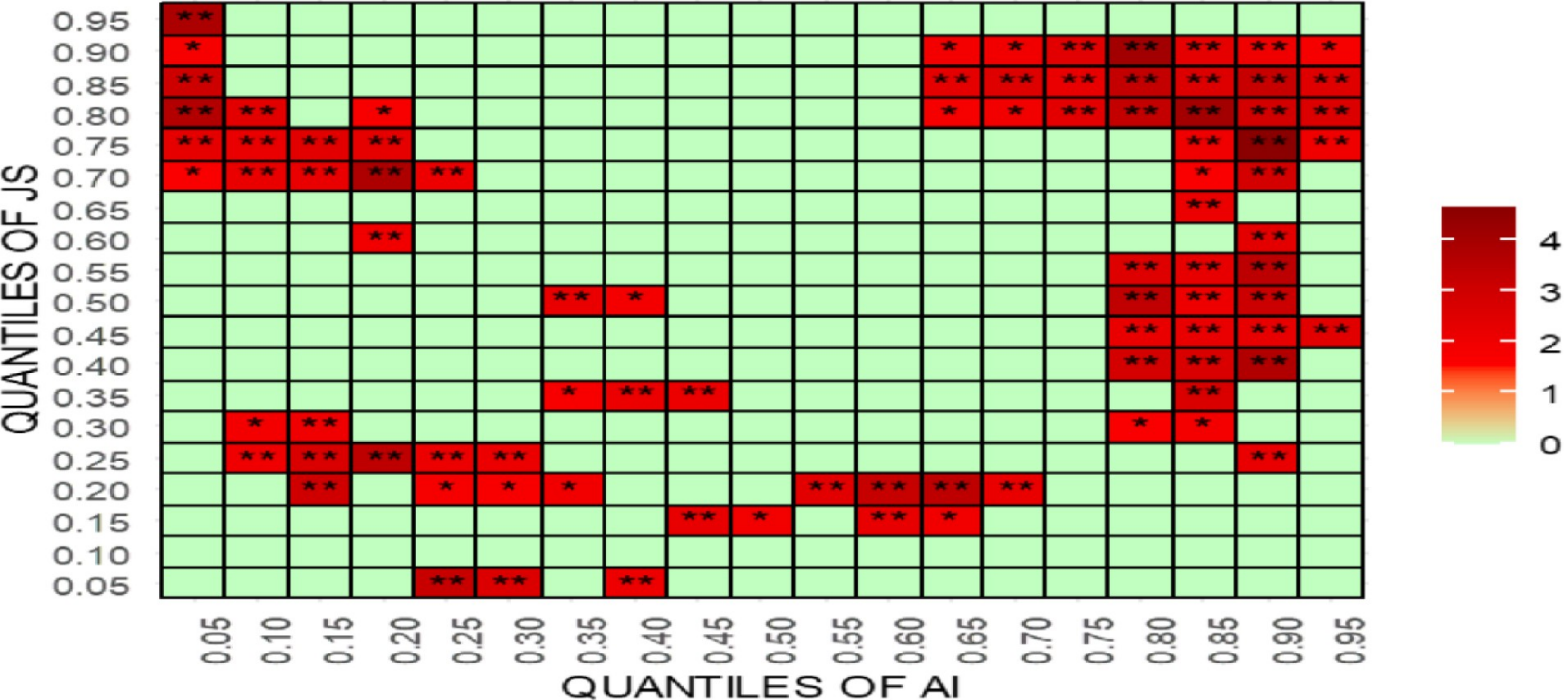
Quantile-quantile Granger causality of AI. This figure presents the results of the quantile-quantile Granger causality test, examining the dynamic relationship between AI and judicial outcomes across different quantiles, highlighting non-linear causal effects over time.

**Fig 28:**
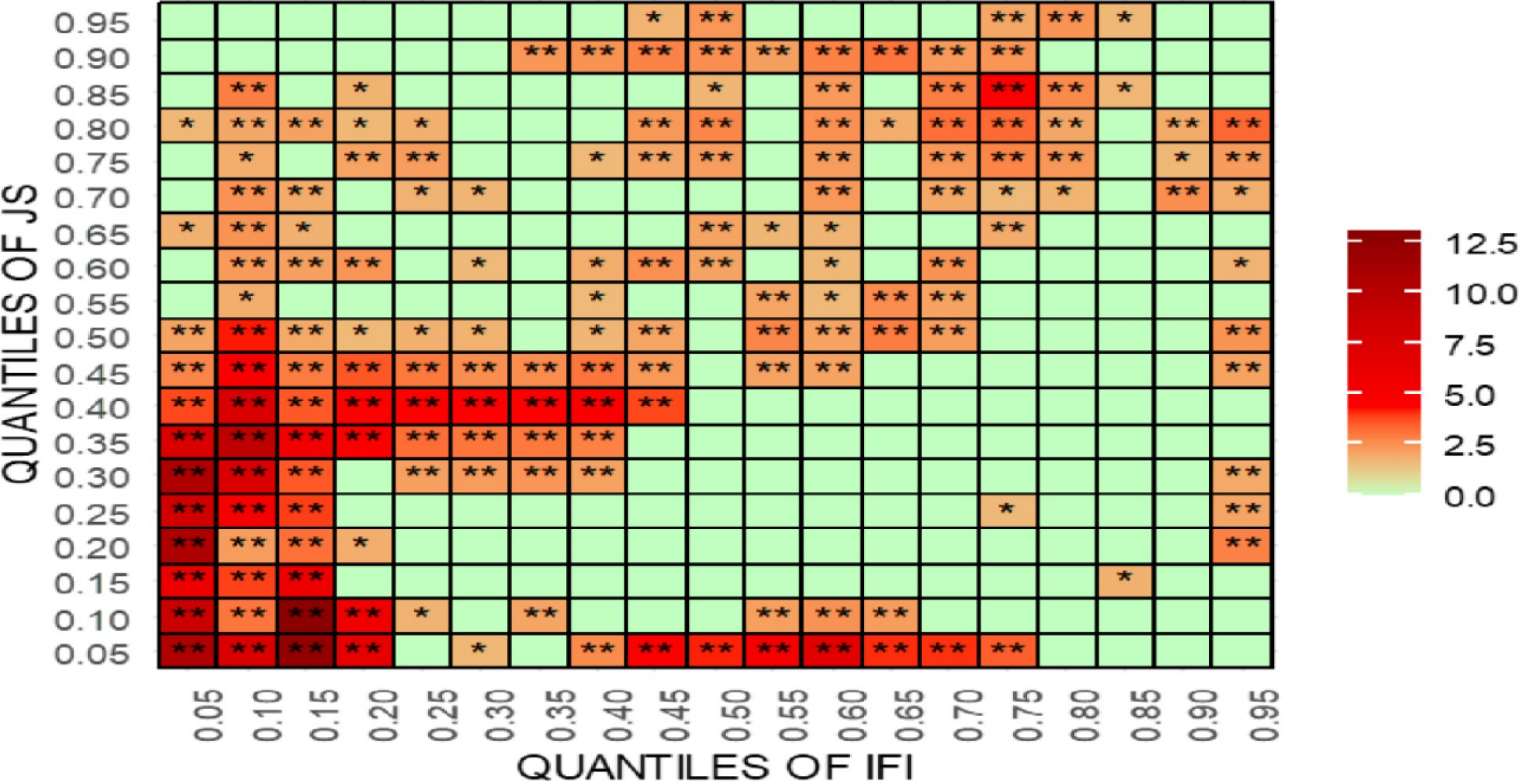
Quantile-quantile Granger causality of IFI. This figure illustrates the results of the quantile-quantile Granger causality test, exploring the dynamic relationship between international financial institutions (IFI) and judicial outcomes across different quantiles, revealing non-linear causal effects.

**Fig 29:**
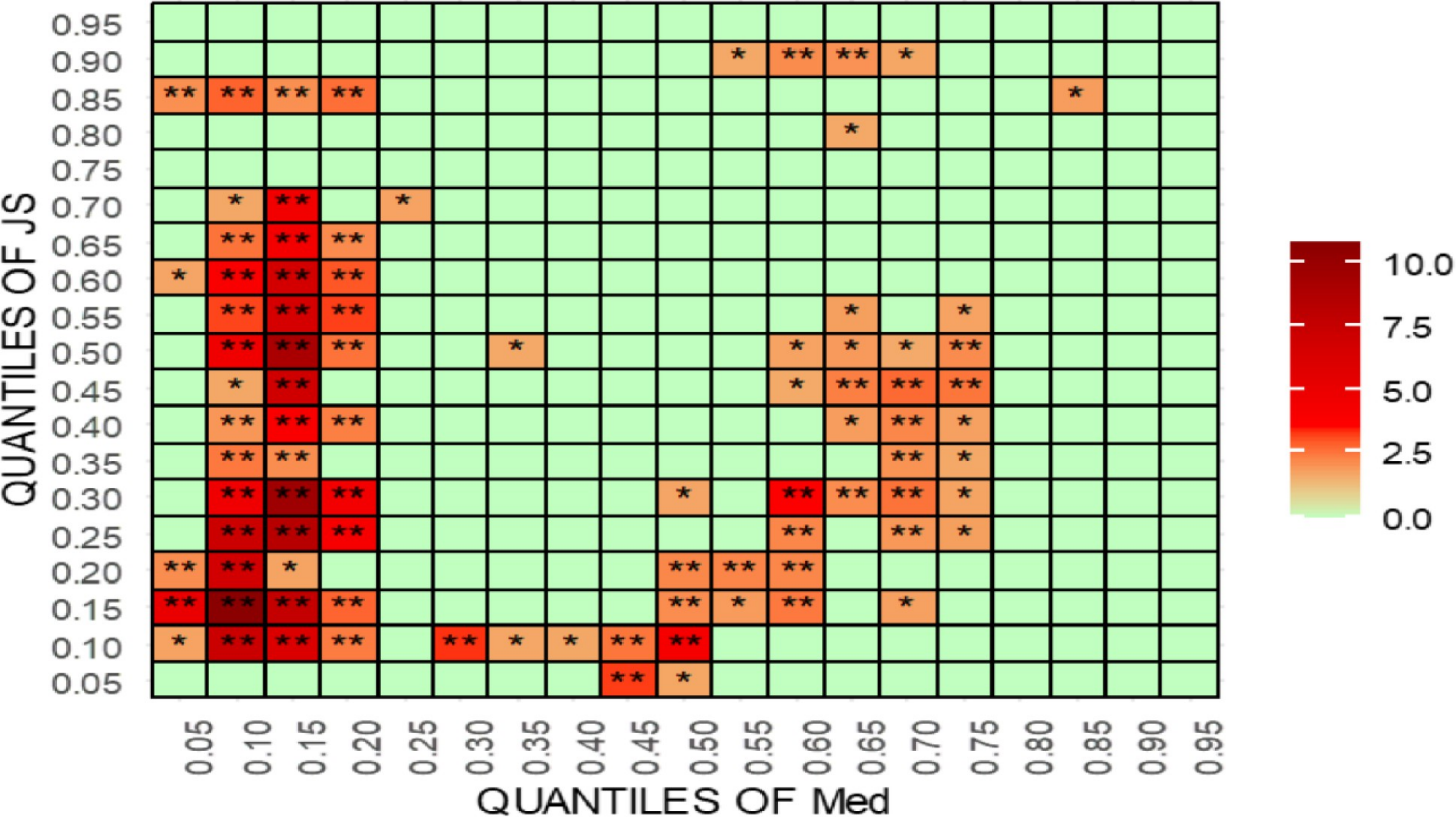
Quantile-quantile Granger causality of media. This figure displays the results of the quantile-quantile Granger causality test, examining the non-linear causal relationship between media influence and judicial outcomes across different quantiles.

**Fig 30:**
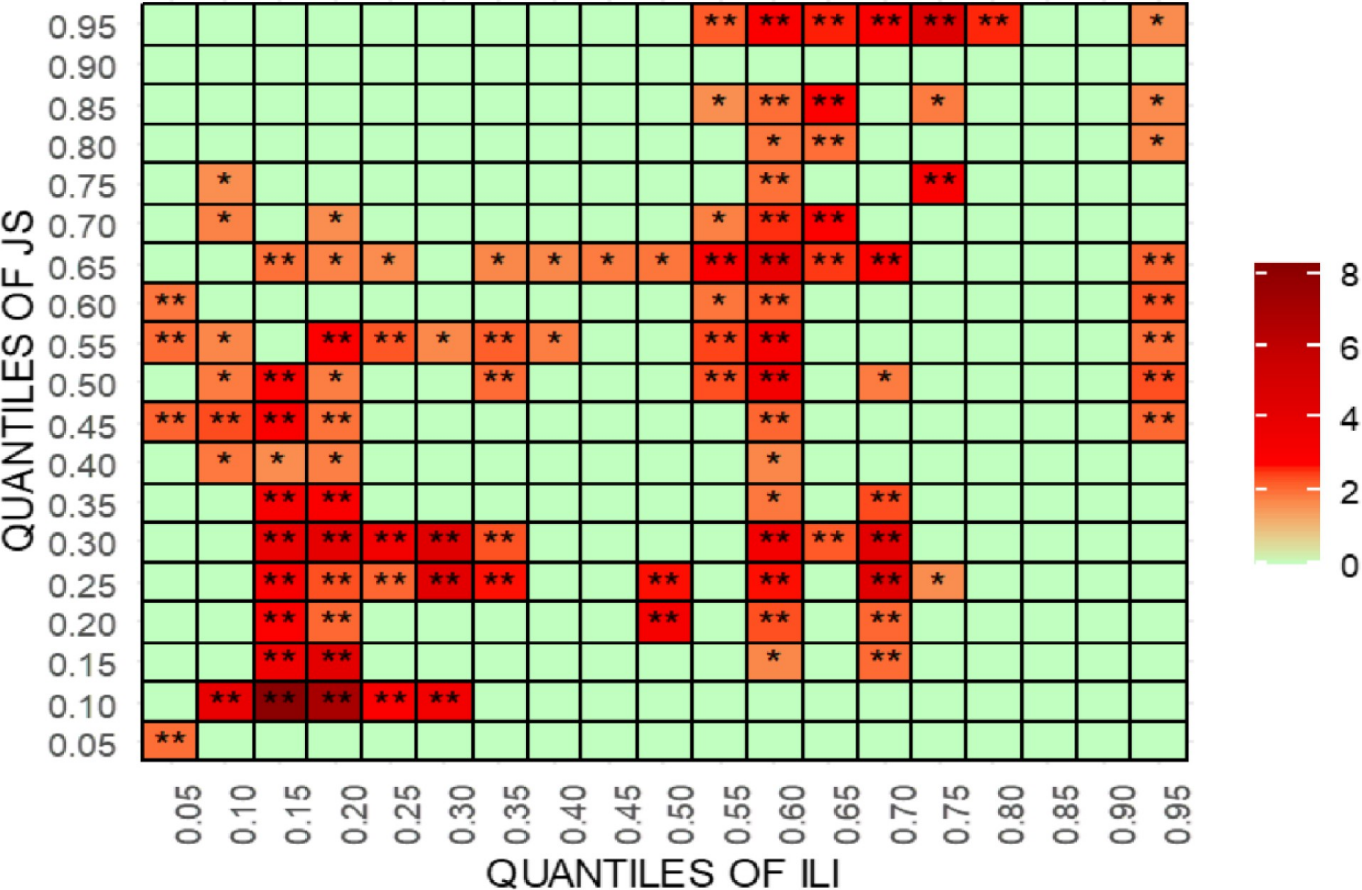
Quantile-quantile Granger causality of ILI. This figure presents the results of the quantile-quantile Granger causality test, analyzing the dynamic relationship between international legal influence (ILI) and judicial outcomes across different quantiles, highlighting non-linear causal effects.

For robustness testing, the article uses [70] new quantile-quantile Granger causality (QQGC). The results are shown in Figs 27–30. Before delving into the results, it is worth mentioning that the QQGC test produces the heatmap results for the 0.05th to 0.95th quantiles. Furthermore, the horizontal axis shows the quantiles for the independent variable, while the vertical axis shows the quantiles for the dependent variable. The colour, like each heatmap, represents the amount of relevance.

Furthermore, the lower quantiles (0.05 to 0.30) represent the short run, the middle quantiles (0.4 to 0.6) represent the medium range, and the higher quantiles (0.7 to 0.95) indicate the long term. Among the several advantages, QQGC reports causality-based outcomes for distinct quantiles, which are classified as short-, medium, and long-run quantiles, allowing for a better understanding of causality levels over different time horizons. Furthermore, the QQGC test can manage nonlinearity difficulties in the selected series. Furthermore, this strategy can mitigate the impact of outliers caused by differences in judicial systems by providing data in different quantiles to help policymakers shape their policies to mitigate bias.

As for the effects of AI shown in Fig 27, the QQGC test confirms that AI significantly granger bias across all time horizons (short to long run). The QQGC test highlights significant causality from AI on judicial bias, implying that policy changes in AI could substantially impact the judicial system across all time horizons. Further, media shown in Fig 28 could not significantly cause judicial system across most of the long-run quantiles; however, it substantially causes bias during the short-run and medium run. The findings imply that changes in IFI have a considerable causal effect on the court system’s performance or bias, particularly in the short and medium term shown in Fig 29. This suggests that fluctuations in IFI may cause immediate and mid-term changes in court system outcomes, potentially affecting policy adjustments in reaction to economic developments. The long-term (higher quantiles) influence of IFI on JS appears to be less apparent, implying that while immediate financial indicators have an impact on the judicial system, these impacts stabilize with time. The same pattern of Granger causality is observed in the case of ILI shown in Fig 30. Based on the outcomes above, it can be inferred that all modelled series significantly Granger cause bias, thereby supporting the study’s main findings. This signifies that any policy changes to these variables could substantially affect judicial system. In summary, all selected series are considerable drivers of economic shocks. Therefore, international authorities should carefully consider the potential response to global economic fluctuations when proposing any policy changes in these variables.

## 6. Discussion

### 6.1 Artificial intelligence innovation

This study primarily focuses on the factors that drive front-end innovation in the judicial system, particularly emphasizing the role of artificial intelligence (AI). The exploration begins with validating a statistical model that has provided significant insights into the judicial system through empirical research. By examining latent constructs and their interrelationships, the indicators have successfully illuminated the connections between variables, showing how dependent variables relate to their respective independent factors. Moreover, the WQC and QQGC analysis confirms that this model reliably represents the component interactions, attesting to its stability and reliability.

Examining the influence of AI on the judicial system (JS), as shown in Fig 3, demonstrates that AI has considerable negative consequences across most quantiles in the short term. This shows that AI may introduce or increase prejudice when used to aid the legal system, especially in the early phases of integration. One possible explanation for this is the initial reliance on existing, potentially biased datasets or algorithms that fail to account for the nuanced nuances of judicial decision-making. During this phase, AI may unintentionally amplify human prejudices or fail to examine contextual aspects critical for impartial verdicts, thus impeding fair case outcomes. Surprisingly, the middle run yields a more complex picture. While AI’s effects are often modest, some higher quantiles demonstrate a favourable impact. This trend suggests that, over time, AI may begin to better match the purposes of the court system, especially in circumstances where a larger volume of data allows AI systems to learn from past cases more efficiently. However, it is important to highlight that this positive influence is not felt equally across all quantiles. This implies that AI’s usefulness may be unevenly distributed depending on criteria such as case complexity, jurisdiction, or the AI system’s specific architecture.

In the long run, AI has a significant potential to improve the judicial process, especially in the lower to medium quantiles, where it allows for more accurate case resolutions with less bias. This trend shows that AI when calibrated and refined over time, could help improve judicial impartiality by providing decision-making tools that take into consideration both legal precedent and fairness. However, this benefit is not ubiquitous. AI appears to be creating bias at higher quantiles when case frequency increases, and case outcomes may be more complex or disputed. This could be owing to the system’s failure to handle the complexities of higher-stakes or more complicated cases, which normally require more human judgment and discretion. Therefore, even though AI has the potential to improve the fairness and accuracy of case resolutions, its long-term efficacy in reducing prejudice is still dependent on a number of variables. These include how AI algorithms continue to advance, what kinds of cases they are utilized for, and how flexible legal systems are in the face of AI-driven procedures. AI may reinforce preexisting prejudices in situations where case complexity or frequency is high, which emphasizes the necessity of constant critical monitoring, openness, and continual learning in AI systems employed in judicial contexts.

The impact of AI on the judicial system, as demonstrated in the findings presented in Fig 3, is significant. Over short, medium, and long terms, AI predominantly exhibits adverse effects across most quantiles, except at lower quantiles in the long run, where it appears to support judicial functions. These negative impacts are categorized into technical, societal, and ethical issues. Initially, AI suffers from quality, bias, privacy, and security concerns in data, which perpetuate existing cognitive biases. Furthermore, the opaqueness of AI algorithms complicates issues of explainability and transparency, demanding substantial resources for training and implementation. Ethically, the lack of a universally accepted framework leads to concerns like undue surveillance and job displacement, exacerbated by the profit-driven motives of large legal tech firms lacking regulatory oversight.

Looking into real cases, the application of AI tools like the COMPAS system highlights these concerns, showing a discernible racial bias in recidivism predictions, with black defendants disproportionately classified as higher risk compared to white defendants. Similarly, AI-generated models such as ChatGPT, while innovative, have demonstrated limitations like producing fictitious legal citations and misinterpreting laws, leading to real-world legal repercussions, as seen in the penalties imposed on attorneys in New York. Despite these challenges, AI also promises significant positive outcomes. Over time, as developers and researchers refine these technologies, their effectiveness and public acceptance increase. Examples include China’s "zhihui fayuan 智慧法院" and Estonia’s use of AI in courts, which demonstrate advancements in legal technology under national policies of digitalization and automation. This agenda has been forwarded by Xi Jinping’s and the Chinese Communist Party’s vision of "yifa zhiguo 依法治国” which means governing the country according to law [33].

Moreover, the capability of AI like ChatGPT to perform highly on bar exams underscores its potential utility in legal analysis. Globally, jurisdictions are becoming more proactive in regulating AI. The European Union’s AI Act, regulations in China, and discussions in the US, UK, and Australia indicate a concerted effort to harness AI’s benefits while mitigating risks. In conclusion, although AI currently presents several challenges to the judicial system, its potential to resolve longstanding issues such as case backlogs, increase judicial efficiency, and reduce biases is promising. Hence, improving algorithmic transparency is crucial; by making AI decision-making processes more accessible and understandable to stakeholders, judicial systems can reduce the risk of hidden biases influencing outcomes. Secondly, human oversight would allow judges and legal professionals to analyze and contextualize AI-generated suggestions, especially in circumstances where sensitive aspects such as demographic data could introduce prejudice. This dual strategy of algorithmic openness and human oversight may prevent AI from making autonomous decisions that are unfair or discriminatory, instead encouraging a balanced usage of AI that promotes equitable justice. With continued technological advancement, regulatory oversight, and increased familiarity and budget allocation, AI can significantly contribute to creating an equitable and unbiased judicial system.

### 6.2 Media and bias

The evaluation of media impacts on the judicial system, as illustrated in the WQC analysis presented in Fig 4, reveals a nuanced influence across different time frames. In the short term, media appears to have an insignificant impact across quantiles. However, the medium term shows a positive shift, with the media supporting the judicial system (JS). In the long term, the influence becomes significantly positive, particularly aiding fair-case conclusions, although some quantiles still show an insignificant impact. It suggests that media could play a crucial role in minimizing bias within the judicial system over time. The positive influence of media is multifaceted, encompassing education of the public, timely dissemination of information, and revealing the broader truths of judicial processes. With the advent of the internet and the proliferation of social media, information is instantaneous and global, enhancing the media’s capability to educate the public on their rights, the legal processes, and the potential consequences of not adhering to laws. Furthermore, attorneys and judges engaging with various media forms help bridge the informational gap between the public and the judicial system.

The media’s role is particularly evident in movements such as "Black Lives Matter" and "Me Too," where it has been pivotal in pushing for reforms by bringing critical issues to the forefront. For instance, the viral media coverage of George Floyd’s murder significantly impacted public discourse and called for urgent judicial and police reforms. During the COVID-19 pandemic, media also played a crucial role in facilitating the continuation of judicial processes through online court coverage and virtual trials, demonstrating its capacity to adapt and maintain essential services. However, the media’s influence is not without its drawbacks. Pre-trial media coverage can adversely affect juror impartiality and influence verdicts. Media outlets often bring high-profile cases into the public eye, potentially altering perceptions and narratives around criminal justice and judicial appointments. It can lead to media-driven narratives that may not always be unbiased, as organizations with specific agendas could fund them. For example, studies have shown that judges may issue harsher sentences influenced by conservative media exposure, and media narratives can polarize public opinion on issues like immigration, contributing to support for restrictive policies.

Moreover, the prevalence of fake news and its role in shaping public opinion against democratic institutions and marginalized groups cannot be understated. For example, racially discriminatory narratives on social media have been linked to increased depression and drug use among young people of colour. The spread of misinformation during the pandemic, dubbed the "Infodemic," has further highlighted the potential harms of unchecked media influence. In summary, while the media predominantly affects the judicial system in ways that can mitigate bias and promote justice, its dual role as both an enabler of transparency and a potential source of bias underscores the complex relationship between media and justice. This dichotomy highlights the need to carefully consider the media’s role in shaping public perception and policy, ensuring it contributes positively to the equitable administration of justice.

Therefore, as a result, the media’s impact on the legal system is multifaceted. Its role in maintaining prejudice cannot be disregarded despite the fact that it has enormous potential to improve fairness and transparency, especially in the medium to long term. As the public and the judiciary adjust to new media dynamics, the media’s short-term, neutral impact may change, but the larger media landscape mostly determines the long-term impacts. The media’s capacity to promote skewed views tempers its capacity to be a force for good, especially in high-profile situations where sensationalism and selective reporting are prioritized.

Additionally, the influence of the media is heavily reliant on the particular legal environment in which it functions. For example, the media might worsen prejudices in well-known instances by swaying public opinion prior to a verdict. However, by educating the public, promoting change, and keeping the judiciary accountable, the media can level the playing field in more common cases or the larger context of judicial reform. Because of this, the relationship between the media and the legal system is intrinsically complicated and necessitates careful evaluation of the framing of media narratives, the dissemination of information, and the degree of accountability and transparency of media institutions.

### 6.3 International legal influence

The impact of international legal influence on the Judicial System (JS), as illustrated in Fig 5, shows a dynamic trajectory from initial adverse effects to substantial long-term benefits. Initially, in the short run, this influence introduces bias into the judicial system, hinting at a complex interplay between domestic and international legal norms. However, as time progresses into the medium term, the influence shifts to provide significant positive assistance, with a few minor exceptions. In the long run, the JS reaps considerable benefits from this international influence, achieving more accurate case conclusions with minimal bias. This overall trend suggests that despite early challenges, international legal influence is advantageous, helping courts refine their case conclusions while reducing inherent biases.

The complexities of this influence are not without their drawbacks. The interjection of international law into municipal systems often creates conflicts due to discrepancies between theoretical frameworks and practical applications. For example, a notable case involved a spy accused of terrorism; although initially sentenced to death, the sentence was overturned by the International Court of Justice, leading to a retrial. This intervention illustrates the sometimes controversial role of international legal bodies in domestic law enforcement. Moreover, developed countries, particularly entities like the European Union, frequently impose legislation on less developed nations. It can disrupt local judicial systems and provoke bias, primarily when the EU uses sanctions independent of United Nations Security Council approval. It reflects contentious international legal practices such as collective countermeasures against violations of universally recognized obligations.

On the positive side, establishing international courts (ICs) where domestic judges collaborate with international counterparts to apply international legal principles within national contexts is a significant development. This model has gained global traction, influencing judicial systems worldwide to adopt similar approaches. Furthermore, The Hague Judgements Convention of 2019 delineates the scope of private and public law, maintaining consistent standards with previous conventions like the 2005 Hague’s Choice of Court Convention. It helps clarify the jurisdictional boundaries and the application of state immunity in international disputes. Another notable advancement is the Legislation of the AI Act, which provides nations with guidelines to manage the complexities of artificial intelligence, emphasizing the importance of international legal standards in shaping domestic policies and practices.

In conclusion, despite some initial adverse effects, international legal influence generally has a positive impact on the judicial system. It guides domestic courts towards alignment with established international norms and supports thorough research and collaboration to ensure just and unbiased decisions. This influence is instrumental in shaping judicial outcomes that are both fair and aligned with global legal standards.

### 6.4 International financial institutions

The impact of international financial institutions on the Judicial System (JS), as depicted in Fig 6, presents a nuanced picture. Initially, in the short term, the influence of these institutions shows mixed results: some lower quantiles experience an insignificant impact, while a minor portion benefits positively. In the medium term, there is a significant shift with positive effects observed across most quantiles, suggesting a robust role in assisting JS in managing court cases effectively. In the long run, the benefits become even more pronounced, with all quantiles experiencing significantly positive outcomes, illustrating a substantial and sustained contribution to accurate case conclusions by international financial institutions. These institutions often impose conditions on loans that require the implementation of legal reforms. These conditions can be controversial, potentially undermining the integrity and sovereignty of national judicial systems. For instance, the International Monetary Fund (IMF) granted a loan to Pakistan contingent upon the privatization of state-owned enterprises and the implementation of specific fiscal policies, showcasing how financial aid is tied to substantial legal and economic restructuring [71].

Despite these complexities, the positive influence of international financial institutions on the judicial system primarily manifests through aid and trade. Institutions like the IMF, the World Bank, and the Asian Development Bank implement various financial aid programs, such as the Financial Sector Assessment Programme (FSAP) and Poverty Reduction Strategy Papers (PRSPs), designed to assist economically disadvantaged nations. These programs support judicial reforms and economic development that indirectly benefit the judicial systems by promoting a more stable and predictable legal environment. Moreover, the success of High-tech Industrial Development Zones (HIDZ) in China, which have significantly contributed to national and local economic expansion, exemplifies the positive impact of external financial support. These zones have thrived due to the substantial economic infrastructure built with the backing of international financial institutions [72].

Furthermore, the correlation between the quality of national institutions and economic prosperity suggests that international financial support enhances economic conditions and bolsters judicial systems by facilitating more secure, efficient, and equitable legal and financial transactions. It is particularly evident in the BRICS nations, where international financial aid has been instrumental in reducing wealth disparity through technological innovation and infrastructure development [73]. In conclusion, while the immediate impact of international financial institutions on judicial systems can be mixed, their long-term influence is predominantly positive, aiding in the delivery of justice and enhancing the overall integrity and capability of judicial systems. This dual role highlights the need for careful consideration of the conditions attached to financial aid, ensuring that the sovereignty of the judicial system is preserved while embracing the benefits that such support can provide.

## 7. Conclusion and policy recommendation

The current study has comprehensively explored the dynamic interactions among the judicial system, artificial intelligence, media, international legal influence, and international financial organizations.

### 7.1 Summary of results

Utilizing the Wavelet Quantile Correlation method, this research has achieved multi-dimensional and robust insights, enabling the formulation of impactful policy recommendations. This approach highlights the interconnectedness of these factors and ensures that the findings are grounded in a method capable of capturing the complexities of their relationships. Table 2 depicts the Wavelet Quantile connection project findings and highlights a significant wavelet-based correlation between variables. Despite the introduction of bias by AI in court cases, AI has the potential to mitigate its own bias and biases within the judicial system, aiding courts in reaching accurate conclusions. Media predominantly influences the judicial system and possesses a solid capacity to diminish bias within it, playing a pivotal role in promoting equitable social justice. Additionally, it can be inferred that while international legal influence may introduce some bias into the judicial system, it also has the potential to reduce and mitigate bias by offering global insights to local systems. Similarly, although international financial institutions may introduce bias in court cases, they can also assist courts in reaching precise conclusions while minimizing bias.

**Table 2:** Summary of the results.

Series	Short-run	Medium-Run	Long-run	Support to SDG-16
**AI**	Mixed	Positive	Positive	✓
**Media**	Mixed but predominantly positive	predominantly positive	Predominantly Positive	✓
**ILI**	Mixed	Positive	Positive	✓
**IFI**	Mixed but predominantly positive	Positive	Positive	✓

Table depicts the results obtained by using wavelet quantile correlation technique for AI, media, ILI, and IFI in short, medium and long–run.

We used new quantile-quantile Granger causality (QQGC) for the robustness test. However, the wavelet quantile correlation approach has inherent sensitivity to non-stationary data. This sensitivity may have an impact on the robustness and reliability of findings, particularly when analyzing medium- to long-term trends in a judicial environment characterized by dynamic and often unpredictable change. Non-stationarity, which occurs when statistical properties such as mean and variance change over time, makes it difficult to accurately capture stable relationships, potentially introducing volatility into the results. As judicial systems evolve, the approach’s reliance on relatively stationary data may limit its ability to capture these changes with high precision, emphasizing the need for complementary methods that account for or mitigate non-stationary influences to improve the robustness of the conclusions reached.

### 7.2 Policy recommendation (Short, medium, and long run)

The study findings provide a robust foundation for policymakers to advance Sustainable Development Goal 16, which focuses on enhancing the judicial system. Based on the Wavelet Quantile Connection Project, as detailed in Table 3, it is possible to draw specific policy recommendations for the short, medium, and long-term periods. These recommendations address the impacts of AI, Media, International Financial Institutions (IFI), and International Legal Influence (ILI) on reducing bias within the judicial system.

**Table 3:** Policy recommendations for AI, media, ILI, and IFI in short, medium, and long-run.

Policy Recommendations	Short-run	Medium-run	Long-run
**AI**	Education Training Pilot Projects	Bias classification Continuous monitoring Data Governance Joint ventures Research & Development	Transparency Accountability Ethical safeguards Legal safeguards
**Media**	Revisit Laws Literacy programs	Continuous monitoring Self-regulation	Enacting Legislation Technology integration
**ILI**	Awareness of the multi-dimensional legal system	Legal Synchronization Public Diplomacy Reciprocity agreements Assistance with the most minor sanctions	Ratification of Treaties Ratification of Conventions
**IFI**	Transparency Institutional reforms Cause vs. profit measurement	Audit Curtailing undue influence Capacity building of countries	Active judicial system International treaties Alternative Financing SDG goals

Table depicts the policy recommendation that can be adopted in short, medium and long run to achieve the goal of SDG -16

Firstly, the integration of AI initially introduces bias due to training on inherently biased data and subjective algorithmic frameworks. Governments must launch extensive educational campaigns to inform the public about the benefits and pitfalls of AI. These should be complemented by training sessions for stakeholders in the judiciary, legal professions, and academia and by partnerships with the tech industry to ensure ethical AI development. Establishing research and development units in collaboration with private tech giants would facilitate better oversight, promote transparency and accountability in AI applications, and ultimately reduce bias in the judicial system. Additionally, the influence of AI on various legal system quantiles should be continuously monitored, with an emphasis on reducing any adverse effects on impartiality. The creation of explainable and contestable AI tools is essential to mitigate the challenge of bias.

Secondly, while the media has generally helped decrease bias in the judiciary, there are instances of undue influence and biased reporting. Especially in high-profile cases, policymakers should make sure that media coverage does not impede court impartiality. This can be accomplished by creating moral standards for covering court cases and encouraging media literacy to raise public awareness of the value of objective reporting in court decisions. Regulations should also be put in place to stop jurors and the public from being influenced by pre-trial media exposure, particularly in politically delicate instances. To address this, the government should revise media laws and encourage ‘self-regulation’ to foster greater transparency. Independent Press Standards Organisation (IPSO) in the United Kingdom and the United States, The News Media Alliance, the Broadcasting Content Complaints Council (BCCC) in India, and media watchdogs such as Reporters Without Borders and the Committee to Protect Journalists (CPJ) are all examples of organizations that promote self-regulation by advocating for greater transparency and ethical media practices. Furthermore, certain news companies, such as the New York Times and the Washington Post, have internal editorial boards that enforce editorial norms that ensure fair and balanced coverage of legal issues. Moreover, a continuous monitoring framework for media coverage of court proceedings should be implemented to ensure balanced reporting. It would prevent the creation of undue hype around AI and ensure a well-informed public.

Thirdly, international legal influences like conventions and legislation by bodies such as the European Union initially introduce bias, which diminishes as domestic systems adapt. It is vital for governments to carefully evaluate international conventions and treaties before integration into local systems to ensure they complement domestic legal frameworks. Public diplomacy and legal synchronization are crucial to mitigate the biases that international legal standards introduce. Lastly, the influence of IFIs, such as the World Bank and IMF, is significant, particularly in underdeveloped countries. While financial assistance is crucial for updating judicial systems and achieving SDG goals, these institutions must operate transparently to avoid undue influence. Governments should enforce policies requiring IFIs to disclose their lending and aid procedures and consider alternative financing options if IFI support introduces bias. The recommendations can be adopted on making priorities as provided by short, medium and long-run.

In order to effectively integrate AI into judicial systems, recommendations can be prioritized and broken down into short- and medium-term action plans. In the immediate term, the emphasis should be on developing AI literacy programs for judges and legal professionals, encouraging transparency in AI-related data, and launching research into bias mitigation in AI algorithms used for judicial purposes. In parallel, building the infrastructure for secure data management platforms and ensuring data transparency will help lay a solid foundation. In the longer term, activities should focus on establishing strong infrastructure to support AI systems, such as regulatory agencies to supervise ethical compliance and the provision of testing environments for AI applications in legal contexts. Furthermore, public awareness efforts on AI’s role in the legal system should be prioritized to foster trust and acceptance. This tiered approach guarantees that critical basic pieces are in place prior to more complex AI integrations, enabling for the effective, equitable, and transparent use of AI in judicial decision-making.

The limitations of this study highlight the necessity for broader research. While the Wavelet Quantile Correlation method has provided valuable insights, other analytical techniques might yield different perspectives or uncover additional factors affecting judicial systems. The scope of data and its limitations also suggest the need for more comprehensive data collection and a broader application of findings across various judicial contexts. Further research could explore the practical implementation of AI in courtrooms, the impact of media coverage on public perceptions, or the effects of specific global events on judicial outcomes. By adhering to these guidelines, policymakers can effectively use the insights from this study to foster a judicial system characterized by fairness, minimal bias, and equitable treatment for all, thus aligning closely with the objectives of Sustainable Development Goal 16.
